# Mesenchymal stem cells and T cells in the formation of Tertiary Lymphoid Structures in Lupus Nephritis

**DOI:** 10.1038/s41598-018-26265-z

**Published:** 2018-05-18

**Authors:** S. Esmaeil Dorraji, Aud-Malin K. Hovd, Premasany Kanapathippillai, Gunnstein Bakland, Gro Østli Eilertsen, Stine L. Figenschau, Kristin A. Fenton

**Affiliations:** 1 RNA and Molecular Pathology Research Group, Institute of Medical Biology, Faculty of Health Sciences, UiT, The Arctic University of Norway, Tromsø, Norway; 2 University Hospital of Northern Norway, Tromsø, Norway; 3 Molecular Inflammatory Research Group, Institute of Clinical Medicine, Faculty of Health Sciences, UiT, The Arctic University of Norway, Tromsø, Norway

## Abstract

Tertiary lymphoid structures (TLS) develop in the kidneys of lupus-prone mice and systemic lupus erythematosus (SLE) patients with lupus nephritis (LN). Here we investigated the presence of mesenchymal stem cells (MSCs) in the development of TLS in murine LN, as well as the role of human MSCs as lymphoid tissue organizer (LTo) cells on the activation of CD4+ T cells from three groups of donors including Healthy, SLE and LN patients. Mesenchymal stem like cells were detected within the pelvic wall and TLS in kidneys of lupus-prone mice. An increase in LTβ, CXCL13, CCL19, VCAM1 and ICAM1 gene expressions were detected during the development of murine LN. Human MSCs stimulated with the pro-inflammatory cytokines TNF-α and IL-1β significantly increased the expression of CCL19, VCAM1, ICAM1, TNF-α, and IL-1β. Stimulated MSCs induced proliferation of CD4^+^ T cells, but an inhibitory effect was observed when in co-culture with non-stimulated MSCs. A contact dependent increase in Th2 and Th17 subsets were observed for T cells from the Healthy group after co-culture with stimulated MSCs. Our data suggest that tissue-specific or/and migratory MSCs could have pivotal roles as LTo cells in accelerating early inflammatory processes and initiating the formation of kidney specific TLS in chronic inflammatory conditions.

## Introduction

Systemic lupus erythematosus (SLE) is an autoimmune disease characterized by systemically deposition of immune complexes (ICs), inflammation and subsequent infiltration of immune cells^1,2^. In lupus nephritis (LN) the deposition of ICs within the glomeruli and tubule interstitial area activate intrinsic kidney and immune cells to produce chemokines attracting more effector cells^3^. Infiltration of immune cells and formation of aggregates in LN are associated with the development of tertiary lymphoid structures (TLS) in both human and murine LN^4,5^.

The formation of TLS is a dynamic process starting with sparse lymphocytic infiltrates that evolve into aggregates and eventually organize into distinct T cell areas and B-cell follicles with germinal centers^6,7^. During secondary lymphoid organ (SLO) development specialized hematopoietic lymphoid tissue inducer (LTi) cells interact with stromal lymphoid tissue organizer (LTo) cells with a mesenchymal origin, in a process involving LTα1β2 signaling to LTβR, which cause LTo cells to express several adhesion molecules (ICAM1, VCAM1, and MAdCAM1) and homeostatic chemokines (CCL19 and CXCL13)^8,9^. The nature and involvement of LTi and LTo cells in the induction of TLS is controversial^8^. In TLS formation, it is believed that immune cells may take the role of LTi cells^10^. Especially the T helper (Th) 17 subset of CD4+ T cells play a central role in the induction of TLS^11^, and various activated resident stromal cells have been shown to be important origin of LTo cells^12,13^. In addition to the known cytokines and chemokines involved in SLO formation, proinflammatory cytokines such as TNF-α and IL-1β have been implicated in the induction and development of TLS^14,15^. High expression of TNF-α in tissue can induce TLS formation in the absence of LTi cells, indicating a role for TNF-α producing myeloid cells^14^. The expression of IL-1β is important in Th17 activation and thus indirectly involved in TLS formation^16^.

Mesenchymal stem cell (MSCs) are adult multipotent progenitor cells, which exist in almost all tissues, and are assumed immunomodulators^17–20^. This function makes them the most prominent therapeutic candidate for autoimmune and inflammatory diseases, as they can inhibit dendritic and T cell proliferation and maturation^21^. On the other hand, based on new evidence, MSCs may have immunostimulatory potentials in specific circumstances, such as low concentration of pro-inflammatory cytokines, and low number of MSCs compared to immune cells in inflamed tissue, which is highly controversial^22,23^. Hitherto, few studies have suggested that MSCs may have immunostimulatory potential. In the review by Ma *et al*. it is suggested that inadequate inflammatory cytokines at chronic inflammatory sites could induce MSCs to produce chemokines and other attracting factors in absence of sufficient immune inhibitory factors^18^. Since MSCs subsist in almost all tissues, their immunostimulatory potentials could play a fundamental role in initiating an inflammatory process in autoimmune diseases. We hypothesize that MSCs play a crucial role as LTo cells, which trigger inflammatory signaling cascade in the kidney that leads to recruitment of immune cells and subsequently TLS formation. We investigated the effect of MSCs on T cell from healthy donors and SLE patients with or without LN. Our results indicate that MSCs in a pro-inflammatory milieu may stimulate CD4+ T cell proliferation and differentiation into Th2 and Th17 effector cells.

## Results

### Mesenchymal stromal-like cells are detected within the kidneys of young lupus-prone mice and within the developed TLS

**Figure 1:**
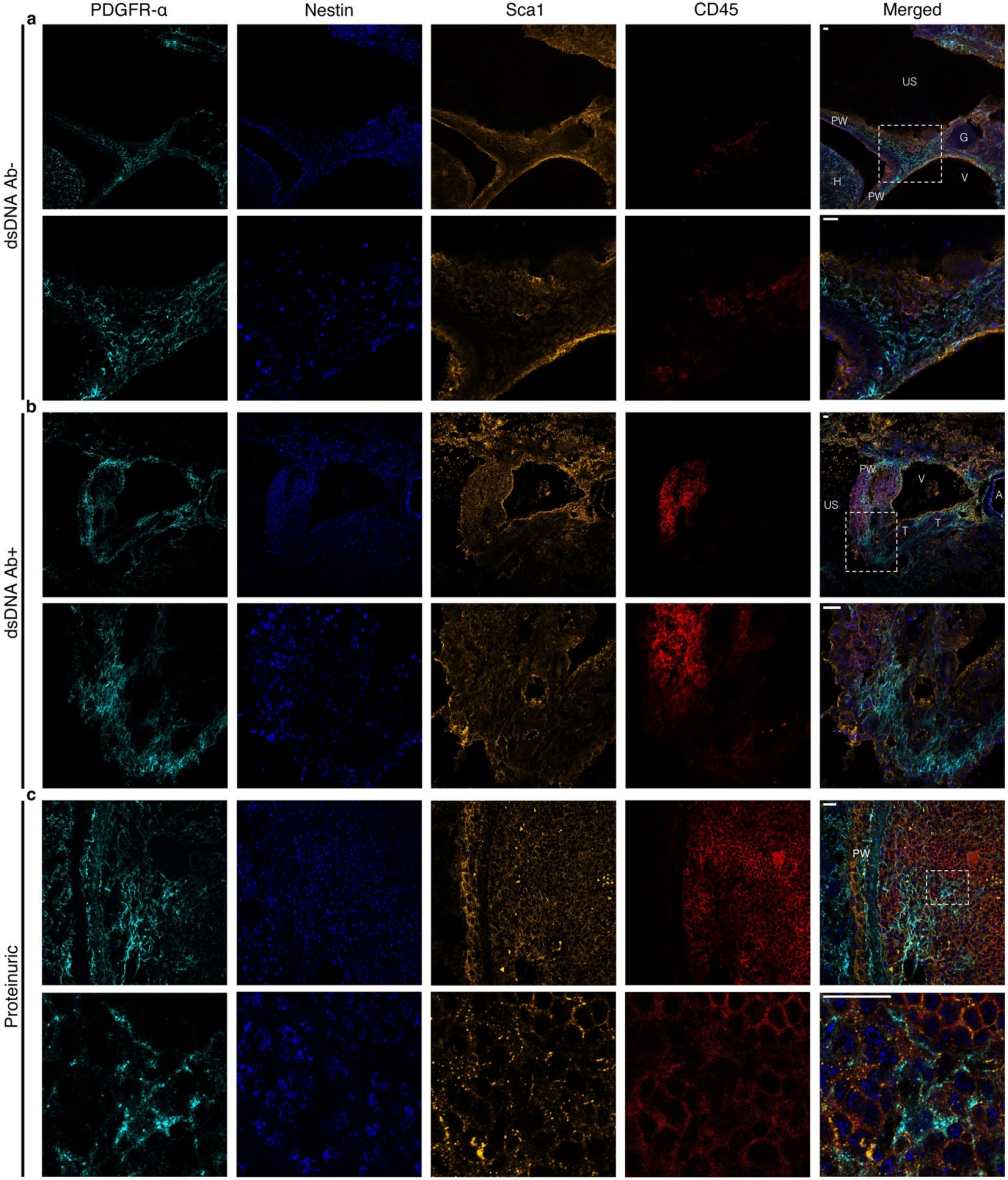
The presence of MSLCs can be detected within the pelvis wall and TLS of lupus-prone mice. MSLCs were detected by using antibodies against Nestin, Sca1, and PDGFRα and the cells were negative selected using antibodies against CD45. Kidney section from (**a**) anti-dsDNA Ab- (n = 3), (**b**) anti-dsDNA Ab+ (n = 3), and (**c**) proteinuric (n = 3) mice were analyzed by IHC. Scale Bar 20 μm. Magnification (**a**–**c**) 20X with digital zoom for magnified panels. (A: artery, Ab: anti-dsDNA antibody, G: Glomeruli, H: Hilum, PW: pelvic wall, T: tubule, US: urinary space, V: vein).

**Figure 2:**
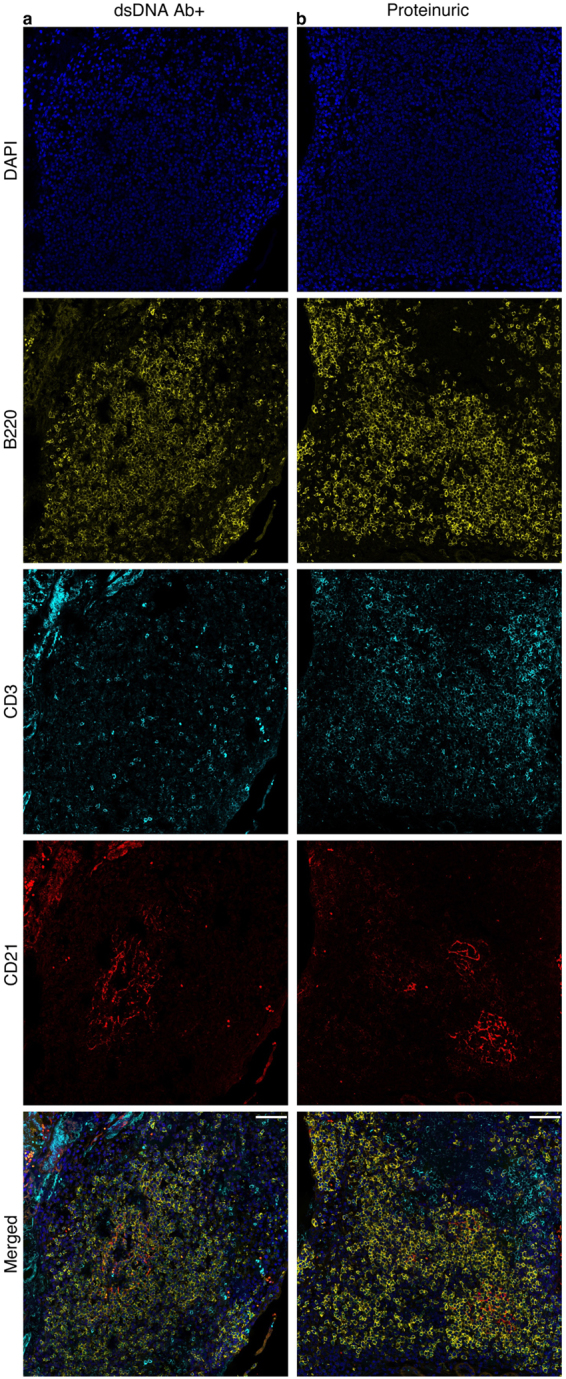
TLS contain germinal centers (GCs) with FDC network and B cells. Kidney sections from (**a**) anti-dsDNA Ab+ mice (n = 10), (**c**) proteinuric (n = 10) mice, and (**d**) BALB/c mice (n = 10) were analyzed for the presence of B cells, T cells, and FDCs using antibodies against B220, CD3, and CD21 respectively, and DAPI to visualize the nucleus. TLS comprising (**a**,**b**) GCs with FDCs supporting networks and distinct T cell zone. Magnification (**a**–**c**) 20X. Scale Bar 20 μm.

Since MSCs play a pivotal role in SLOs function, organization, and tissue homeostasis and SLOs share similarity with TLS in function and structure^24,25^, we investigated the localization of mesenchymal stem-like cells (MSLCs) in kidneys of lupus-prone mice. In young Ab− mice (6 wo), Nestin^+^Sca1^+^PDGFRα^+^CD45^−^ MSLCs were mostly located in the pelvic wall, and CD45+ tissue-residential immune cells were located at the edge of the pelvic wall surrounded by MSLCs (Fig. 1a). In older Ab+ (30 wo)and proteinuric (27 wo) mice the Nestin^+^Sca1^+^PDGFRα^+^CD45^−^ MSLCs were detected in the pelvic wall around and within the TLS and adjacent to CD45+ immune cells with a lower ratio (Fig. 1b,c, respectively). The signal intensity graphs indicate that the areas positive for PDGFRα were negative for CD45 and contrariwise (Supplementary Fig. S1). We also observed GCs with supporting FDC network and distinct T cell zones in Ab+ and proteinuric lupus-prone mice (Fig. 2)

**Figure 3:**
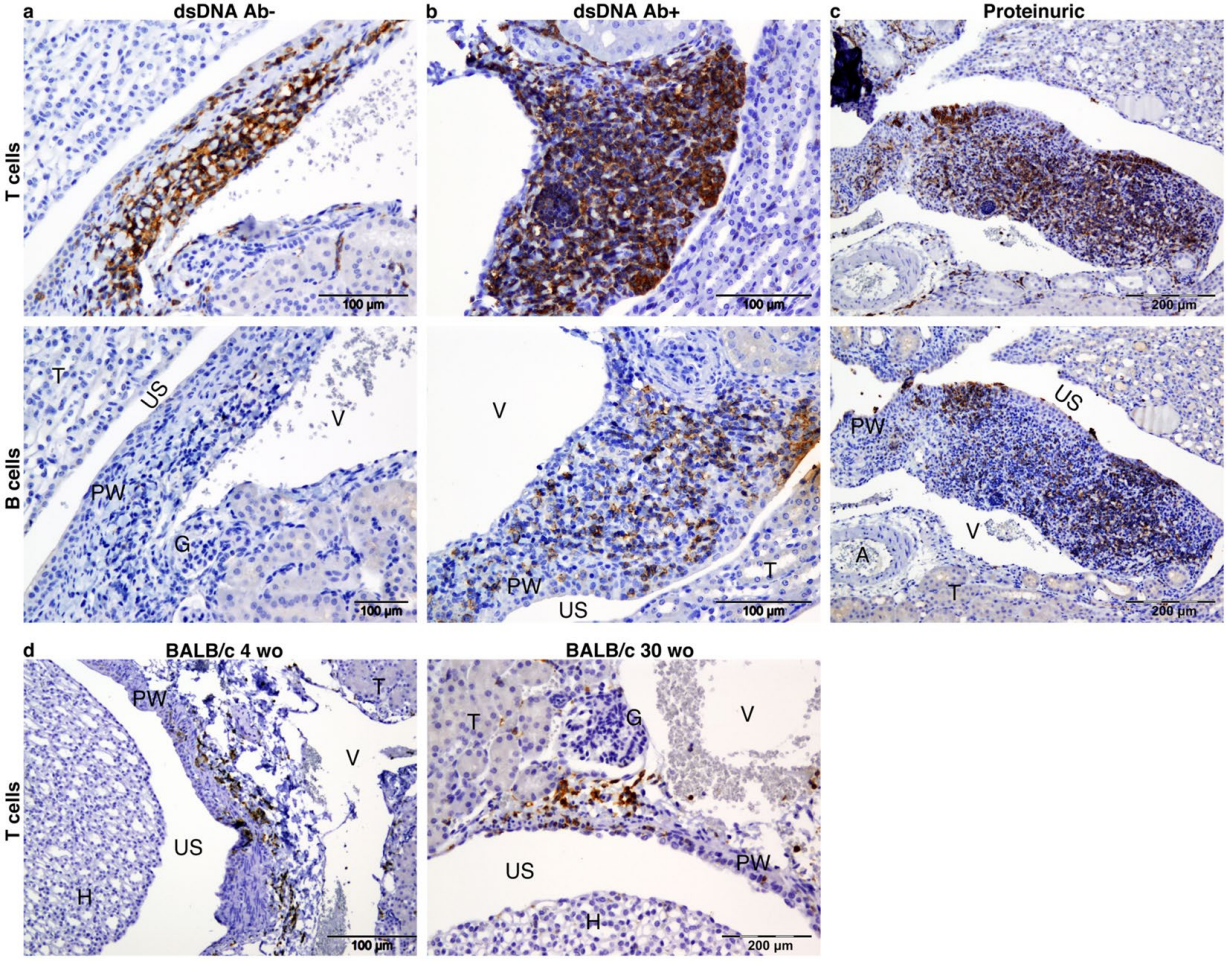
T and B cell are accumulating within the pelvic wall during the formation of TLS. Kidney sections from (**a**) young anti-dsDNA Ab- mice (n = 24), (**b**) anti-dsDNA Ab+ (n = 27), and (**c**) proteinuric (n = 27) mice were analyzed for the presence of T and B cells using antibodies against CD3 and B220, respectively. Total RNA was isolated from NZB/W mice at different ages. (A: artery, Ab: anti-dsDNA antibody, G: Glomeruli, H: Hilum, PW: pelvic wall, T: tubule, US: urinary space, V: vein).

**Figure 4:**
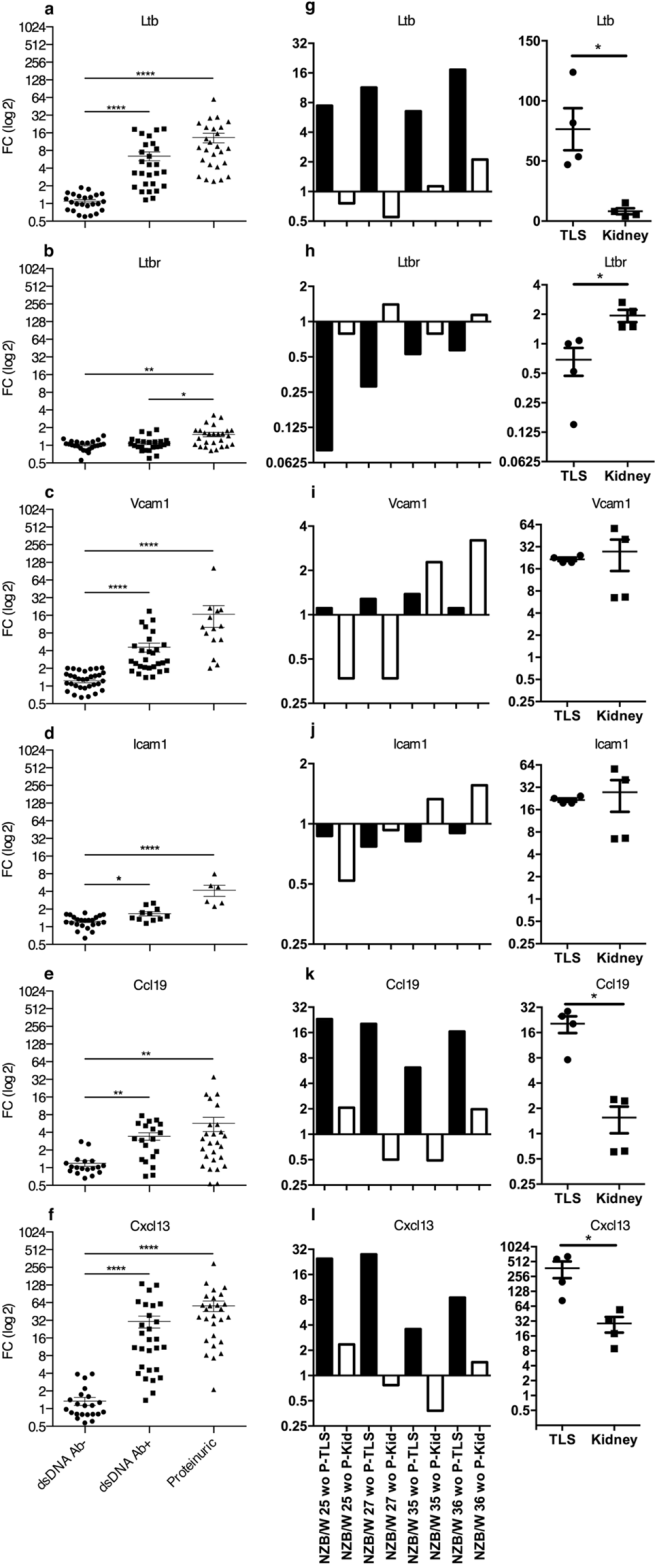
Cytokines and chemokines attracting T and B cells are upregulated in kidneys during development of TLS and nephritis. The total kidney gene expression of (**a**) LTβ (n = 24, 27 and 26), (**b**) LTβR (n = 24, 24 and 26), (**c**) VCAM1 (n = 35, 28 and 14), (**d**) ICAM1 (n = 23, 11 and 6), (**e**) CCL19 (n = 18, 19 and 26) (**f**) CXCL13 (n = 24, 28 and 27) were analyzed by qPCR. mRNA expressions are shown as FC mean ± SEM compared to young 7 wo mice. Kruskal-Wallis test with Dunn’s Multiple Comparison Test. *P < 0.05; **P < 0.01; ****P < 0.0001. Gene expression in isolated TLS and kidney tissue from proteinuric mice was normalized against average ΔCT of all kidney tissue samples (**g**–**l**). The gene expression in TLS and kidney tissue was also normalized to young 7 wo mice (second panel in g–l).

The renal pelvis areas of these mice were also evaluated for the presence of T and B cells (Fig. 3). Residential T cells were found in the pelvis wall of young Ab- mice. However, B cells were not detected in these areas (Fig. 3a). In Ab+ + mice the majority of the immune cells within TLS were T cells (Fig. 3b), whereas, in proteinuric mice with extensive TLS formation, there were more B cells. The majority of cells were still T cells, but at a lower ratio compared to Ab+ mice (Fig. 3c). The expression of cytokines and chemokines essential for TLS development in kidneys of NZB/W was detected at different ages. The mRNA expression of Ltb, Vcam1, Icam1, Ccl19, and Cxcl13 were significantly increased during disease progression in the kidneys of 18 weeks old (wo) and older anti-dsDNA Ab+ NZB/W mice (Fig. 4a,c–f, respectively). The expression of LTβR seemed to be unchanged although a significant increase was observed in the proteinuric mice of age 23–39 wo (Fig. 4b). To measure the expression of these genes within the TLS compared to the kidney expression, RNA were isolated from TLS and kidney tissue dissected from 25–36 wo proteinuric NZB/W mice and analyzed by qPCR (Fig. 4g–l). The results revealed an increased expression of Ltb, Ccl19 and Cxcl13 in TLS compared to the kidney (Fig. 4g,k and l). The expression of Ltbr was increased in kidneys compared to TLS (Fig. 4h).

### Human MSCs express TNF-α, IL-1β, CCL19 and ICAM1 upon stimulation with inflammatory cytokines

**Figure 5:**
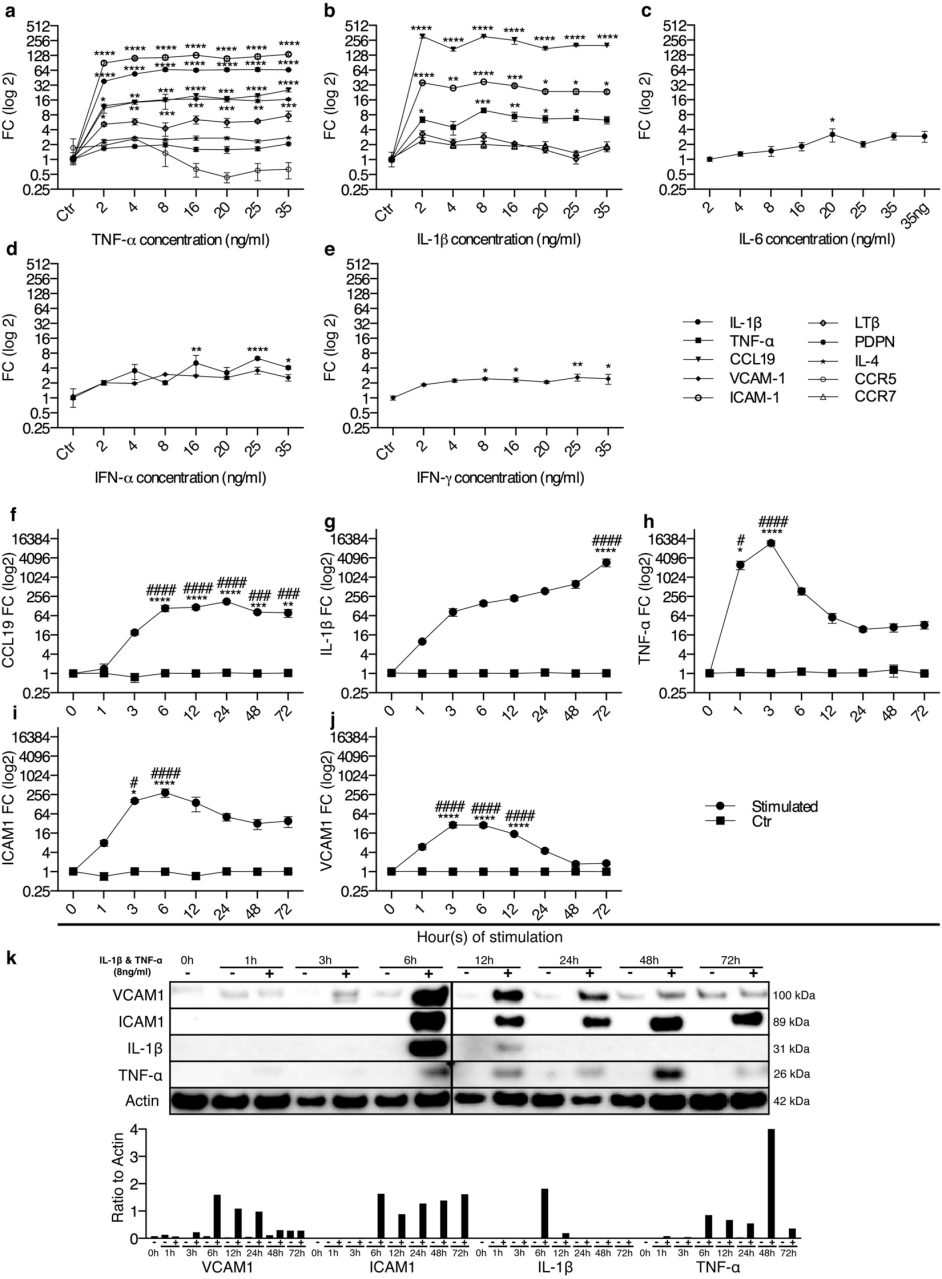
Augmentation of proinflammatory cytokines and adhesion proteins in MSCs upon stimulation with TNF-α and IL-1β. MSCs (n = 3) were stimulated for 24 h with different concentrations of (**a**) TNF-α (**b**) IL-1β (**c**) IL-6 (**d**) IFN-α and (**e**) INF-γ. mRNA expressions are shown as FC mean ± SEM compared to control (Ctr). MSCs (n = 3) were stimulated with a combination of IL-1β and TNF-α (8 ng/ml) and mRNA expression of (**f**) CCL19, (**g**) IL-1β, (**h**) TNF-α, (**i**) ICAM1, and (**j**) VCAM1 were measured after 1–72 h. Results are shown as FC mean ± SEM compared to Ctr at the same time point. (**k**) The protein expression of MSCs were analyzed by Western blotting using antibodies against VCAM1, ICAM1, IL-1β, TNF-α and Actin. The quantitative ratio to actin was measured (k). (**a**–**e**) Two-way ANOVA with Post-hoc analysis Dunnett’s multiple comparisons test within a group toward Ctr (*). (**f**–**j**) Two-way ANOVA with Post-hoc analysis Sidak’s (compare between the groups Ctr and stimulated at given time point, marked with *) and Dunnett’s (compare within a group toward zero hour time point, marked with #) multiple comparisons tests. *^/#^P < 0.05; **^/##^P < 0.01; ***^/###^P < 0.001; ****^/####^P < 0.0001.

We evaluated the MSCs response to an inflammatory and anti-inflammatory environment by stimulating the cells with different cytokines (Fig. 5 and Supplementary Tables S1 and S2). MSCs stimulated with TNF-α or IL-1β expressed higher levels of CCL19 and ICAM1 compared to unstimulated controls (Fig. 5a,b). TNF-α, IL-6 or INF-α induced IL-1β expression (Fig. 5a,c,d), whereas IL-1β or IFN-γ resulted in higher levels of VCAM1 (Fig. 5b,e), and IL-1β increased the expression of TNF-α in MSCs (Fig. 5b). The anti-inflammatory cytokine IL-10 did not induce any significant increase or decrease in the gene expression of the analyzed genes (Supplementary Table S2). Unstimulated MSCs (nsMSCs) expressed high basal levels of VCAM1, ICAM1, LTβR, CXCL12, and lower levels of IL-1β, PDPN, and IL-7 (Supplementary Tables S1 and S2).

The inflammatory property of MSCs was further assessed by dual IL-1β and TNF-α stimulation. Stimulated MSCs (sMSCs) expressed higher mRNA levels of CCL19, IL-1β, TNF-α, ICAM1, and VCAM1 compared to the controls and to nsMSCs at zero hour (Fig. 5f–j). At the protein level, the expression of VCAM1, ICAM1, IL-1β, and TNFα was detected in sMSCs (Fig. 5k). Other candidates for LTo cells are endothelial and epithelial cells. To investigate if such cells responded similar to cytokines as the MSCs, endothelial (HUV-EC_C) and epithelial cells (HMLE) were stimulated with IL-1β and TNF-α. These cells showed similar expression pattern as sMSCs (Supplementary Fig. S2A–G). However, no expression of CCL19 and CXCL13 were observed.

### CD4+ T cells proliferate in co-culture with MSCs

**Figure 6:**
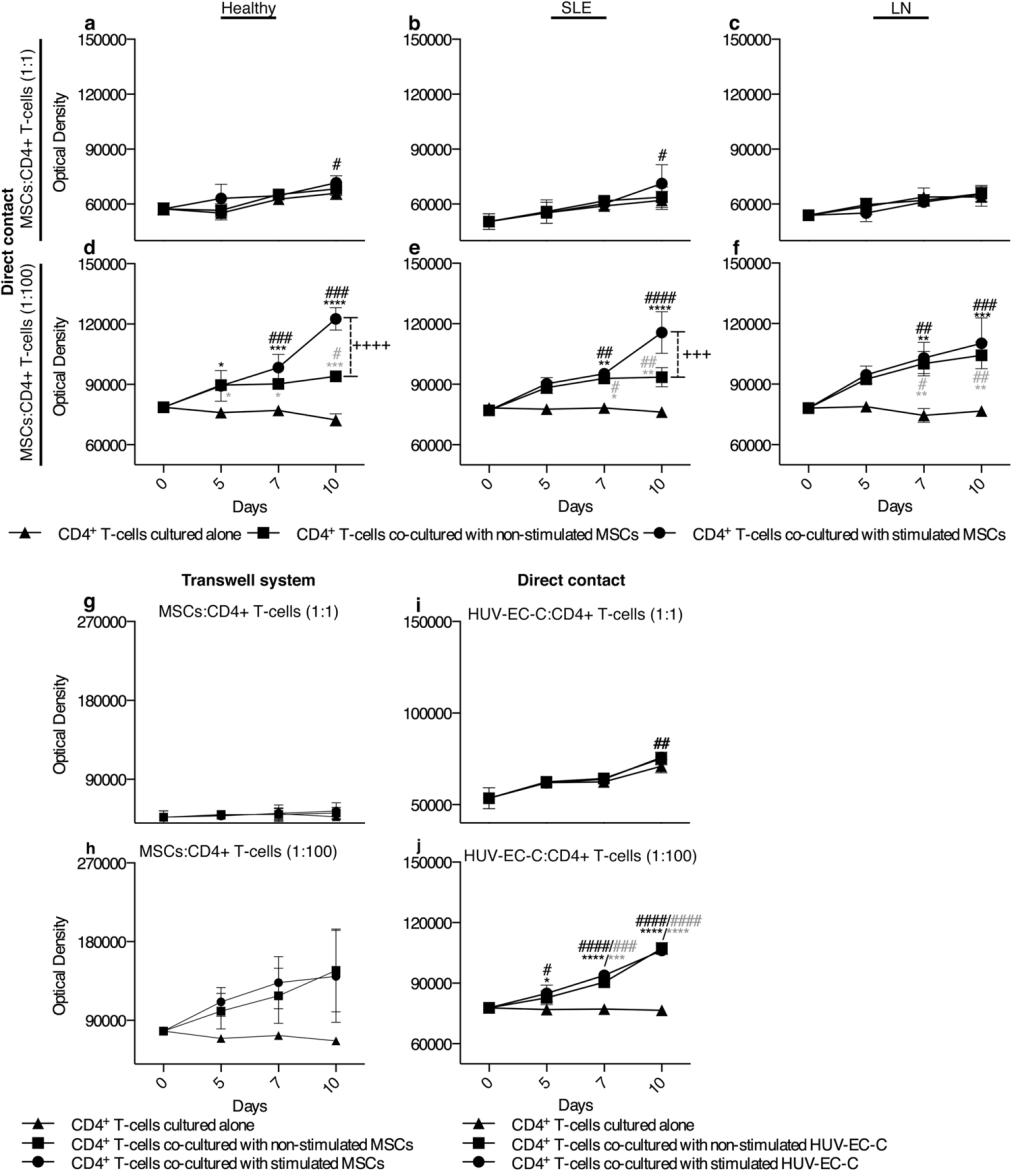
CD4+ T cells proliferate more at high ratio in co-cultured with sMSCs. CD4+ T cells from (**a**,**d**) Healthy (n = 5), (**b**,**e**) SLE (n = 5) and (**c**,**f**) LN (n = 5) were co-cultured with sMSCs at (**a**–**c**) 1:1 and (**d**–**f**) 1:100 ratio (MSCs:T cells) and proliferation was measured at day 5, 7 and 10. To investigate if the proliferation was contact dependent, CD4+ T cells from a Healthy donors (n = 2) were co-cultured with sMSCs and nsMSCs, or cultured alone at (**g**) 1:1 and (**h**) 1:100 ratio (MSCs: CD4+ T cells) in a Transwell system. Proliferation was measured at day 5, 7 and 10. CD4+ T cells from (**i,j**) Healthy (n = 3) were co-cultured with sHUV and nsHUV at 1:1 (**i**) and 1:100 ratio (**j**) and proliferation was measured at day 5, 7 and 10. Data are shown as optical density mean ± SEM. Two-way ANOVA and with post-hoc analyses Dunnett’s (compare different days within a group toward day zero, marked with #) and Tukey’s (compare stimulated and non-stimulated setups toward CD4+ T cells cultured alone at a given time point, marked with * and compare stimulated setup toward non-stimulated setup at a given time point, marked with+) multiple comparison tests. *^/#^P < 0.05; **^/##^P < 0.01; ***^/###^P < 0.001; ****^/####^P < 0.0001. (*^/#^co-cultured with sMSCs or sHUV, *^/#^co-cultured with nsMSCs or nsHUV).

In the induction and expansion of TLS activated organizer cells will recruit naïve T cells. We therefor investigated the proliferative response of CD4+ T cells isolated from peripheral blood of healthy donors, SLE, and LN patients (Supplementary Table S3) by co-culturing the cells with sMSCs or nsMSCs. No clear increase in T cell proliferations at 1:1 co-culture were observed after ten days (Fig. 6a–c). However, the presence of sMCSs and nsMSCs in co-culture at 1:100 ratio initiated a proliferative response of CD4+ T cells, compared to T cells cultured alone (Fig. 6d–f). In addition, co-culture with sMSCs induced a significantly higher proliferation of T cells in the Healthy and SLE group compared to nsMCSs, indicating the importance of stimulation of MSCs upon inducing T cell proliferation (Fig. 6d,e). However, this was not observed for T cells from the LN group (Fig. 6f) and neither for healthy donors in a Transwell system (Fig. 6g,h), suggesting a contact dependent inhibition of T cell proliferation by nsMSC for the Healthy and SLE group (Fig. 6d,e). Interestingly, there was no significant difference between CD4+ T cells co-cultured with non-stimulated HUV-EC-C (nsHUVs) and stimulated HUVs (sHUVs) on day ten at 1:1 or 1:100 ratio, indicating no contact dependent inhibition of T cell proliferation by HUVs (Fig. 6i,j).

### Healthy CD4+ T cells differentiate in co-culture with stimulated MSCs in direct contact

**Figure 7:**
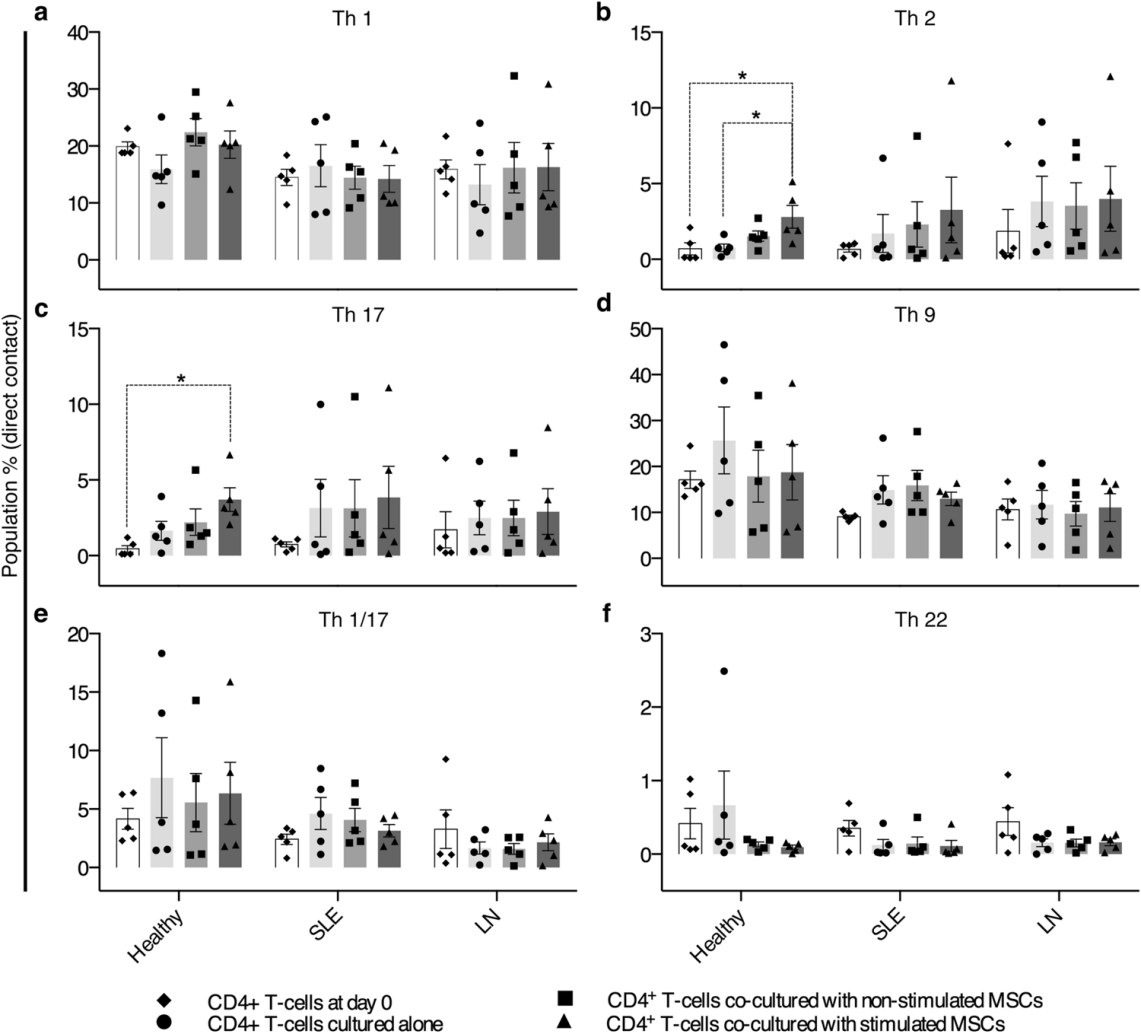
CD4+ T cells from the Healthy group differentiate into Th2 and Th17 subsets in direct co-culture with sMSCs. After co-culturing at 1:1 ratio and 1:100 ratio with nsMSCs and sMSCs or cultured alone, CD4+ T cells from Healthy, SLE and LN groups were analyzed by FACS. The T cells co-cultured 1:100 with nsMSCs, sMSCs or cultured alone were analyzed for (**a**) Th1, (**b**) Th2, (**c**) Th17, (**d**) Th9, (**e**) Th1/17 and (**f**) Th22. Data are shown as % population mean ± SEM. One-way ANOVA with Post-hoc analysis Sidak’s multiple comparisons test (compare within groups, marked with *). *P < 0.05.

To evaluate the immune-regulatory potential of MSCs on CD4+ T cell differentiation into the T helper cell subsets, sMSCs and nsMSCs were co-cultured with T cells from donors (Supplementary Table S3, Supplementary Fig. S3). CD4+ T cells isolated from the Healthy group co-cultured with sMSCs at 1:100 ratio had increased Th2 and Th17 subsets compared to day zero (Fig. 7b,c). There were no difference in the Th1, Th9, Th1/17 and Th22 between the setups and groups (Fig. 7a,d,e and f). Using the Transwell system we observed no difference between the setups within the groups (Supplementary Fig. S4), which indicates a contact dependent differentiation of the Th2 and Th17 cells observed in Fig. 7b,c. No differences in the Th subsets were observed in T cells isolated from SLE and LN when co-cultured with nsMSCs and sMSCs (Fig. 7). The different Th subsets and the % of live CD4+ T cell populations measured at day 0 are listed in Supplementary Fig. S3.

### MSCs co-cultured with T cells express CXCL13

**Figure 8:**
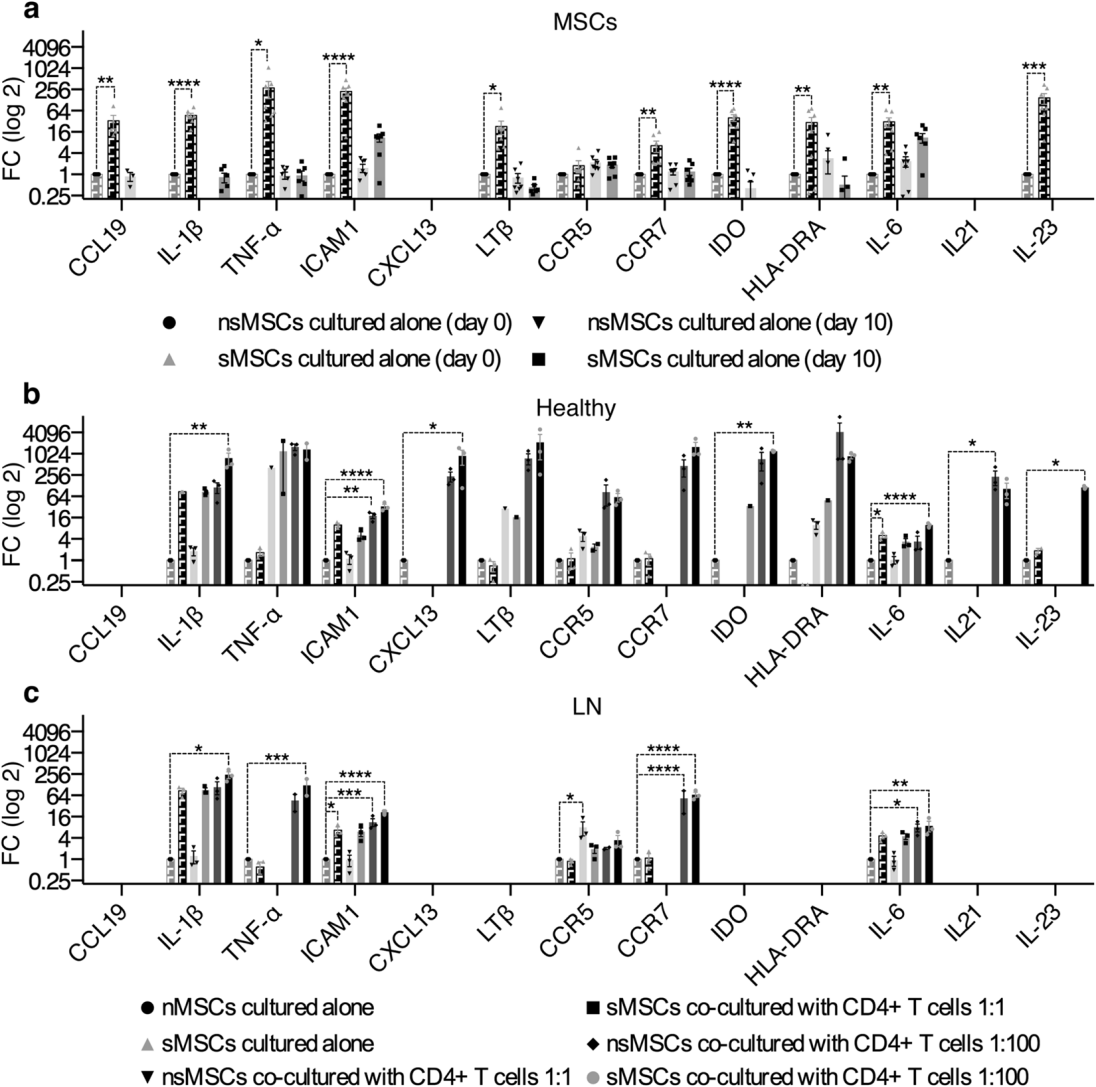
Stimulated MSCs express higher levels of homeostatic cytokines and chemokines in co-culture with CD4+ T cells from the Healthy group. (**a**) MSCs (n = 7) were stimulated for 6 h with IL-1β and TNF-α (8 ng/ml) and analyzed for the mRNA expression of CCL19, IL-1β, TNF-α, ICAM1, CXCL13, LTβ, CCR5, CCR7, IDO, HLA-DRA, IL-6, IL-21 and IL-23 at day 0 and day 10. Results are given as FC mean ± SEM compared to nsMSCs at day 0. (**b**) nsMSCs and sMSCs in co-culture with CD4+ T cells at 1:100 ratio from the Healthy group (n = 3). Results are given as FC mean ± SEM compared to nsMSCs at day 10. (**c**) nsMSCs and sMSCs in co-culture with CD4+ T cells at 1:100 ratio from the LN group (n = 3). Results are given as FC mean ± SEM compared to nsMSCs at day 10. (One-way ANOVA with Post-hoc analysis Dunnett’s multiple comparisons test (compared within a group toward nsMSCs at given time point, marked with *). *P < 0.05; **P < 0.01; ***P < 0.001; ****P < 0.0001.

We further examined the effect of co-culturing CD4+ T cells from the Healthy and LN group with MSCs on the expression of cytokines, chemokines and adhesion molecules. sMSCs expressed significantly higher mRNA levels of LTβ, CCR7, IDO, HLA-DRA, IL-6 and IL-23 compared to nsMSCs on day zero (Fig. 8a). CD4+ T cells from the Healthy group induced expression of CXCL13, IDO, IL-21, and IL-23 in sMSCs compared to nsMSCs cultured alone (Fig. 8b). However, in sMSCs co-cultured with T cells from the LN group (n = 3, patients 8, 9 and 10, Supplementary Table S3) no expression of CCL19, CXCL13, IDO, HLA-DRA, IL-21, and IL-23 were detected (Fig. 8c). This may indicate that the T cells from the LN group inhibit the expression of these genes. sMSCs co-cultured with CD4+ T cells from both groups expressed significantly higher levels of IL-1β, ICAM1, CCR7, and IL-6 compared to nsMSCs cultured alone (Fig. 8b,c). sMSCs and nsMSCs in co-culture with CD4+ T cells from LN group expressed higher levels of ICAM1, CCR7, and IL-6 indicating an activation of nsMSCs by the T cells (Fig. 8c). Interestingly, the expression pattern observed in nsHUVs and sHUVs after co-culture with CD4+ T cells isolated from peripheral blood of Healthy donors (n = 3), were similar as observed for nsMSCs and sMSCs (Supplementary Fig. S2).

## Discussion

In an autoimmune scenery, the constant exposure of autoantigens will lead to the persisting activation of immune cells and subsequent development of TLS^26^. These processes increase the inflammatory cascade by providing local activation of immune cells and production of autoantibodies. TLS formation within tissues undergoing chronic inflammation requires cross talk between stromal cells as LTo cells and resident or recruited immune cells as LTi cells^7,26,27^. Persistent exposure to ectopic expression of proinflammatory cytokines causes fundamental changes in the stromal cells and lead to the expression of homeostatic chemokines and adhesion molecules, which result in recruiting, organizing and aberrant accumulation of immune cells^5,13,27–34^. However, it is still unclear whether MSCs can act as LTo cells in development of kidney-specific TLS, but our results suggest that resident tissue-specific MSCs could potentially reprogram as lymphoid like stromal cell in inflammatory milieu and act as LTo cells through interaction with T cells as LTi cells induce TLS formation and start the early inflammatory cascade. More important, the MSCs produced chemokines attracting naïve T cells, and unstimulated T cells proliferated and differentiated when co-cultured with sMSCs.

We have previously demonstrated that IL-1β overexpression in lupus-prone mice is detectable at an early stage of disease^35,36^, and it has been shown that activation of the TNF-RI-signaling pathway is sufficient to induce upregulation of adhesion molecules and homeostatic chemokines, and TLS formation^13,14,31,37^. Peduto *et al*. were the first to demonstrate that stromal cells undergo changes at the site of inflammation^38^. They suggested that tissue-specific stromal cells, under inflammatory conditions, reprogram as “lymphoid” stromal cells. This gave them the competence to express homeostatic chemokines required for immune cells recruitment and maintenance as has been observed in lymphoid organs^38,39^(1,2). These gp38^+^ “lymphoid” stromal cells could be generated from residential fibroblasts or mesenchymal precursors in inflamed tissue^38^(1). It has been shown that LTo cells in the lymph node express the mesenchymal lineage markers PDGFRα and PDGFRβ, and kidney-specific mesenchymal cells are PDGFRα posetive^40–42^. Koning *et al*. reported that the majority of lymph node stromal cell subsets are Nestin^+^ MSCs lineage. Nestin^+^ bone marrow MSCs could give rise to CD31^−^gp38^+^ mesenchymal and CD31^+^gp38^+^ endothelial LTo cells, which both play a crucial role in lymph node development^43^. This is in agreement with our results as we detected Nestin^+^Sca1^+^PDGFRα^+^CD45^−^ MSCs and T cells in the pelvis wall of autoantibody negative lupus-prone mice. In addition, We demonstrated that kidney-specific TLS comprise T cell areas and GC like structures with supporting FDCs network in autoantibody positive and proteinuric mice. If MSCs are originating from kidneys itself or if they are migrating from the bone marrow are not know. Consequently, MSCs can differentiate into other stromal cells, through interaction with CD4+ T cells and activation through TNF-receptor signaling, provided by early inflammatory stimuli or B cells^28,37,44^. Thus, aberrant immune cell accumulation and MSCs differentiation leads to enlargement of lymphatic networks and vessels, and the expansion of TLS^45^.

Several studies have shown that MSCs possess two distinct regulatory potential depending on the MSCs ratio that could inhibit or promote T cell proliferation^32–34^. Blanc *et al*. showed that high number of MSCs resulted in suppression of T cell activity, whereas an enhancement of proliferation was observed at the low number of MSCs^33^. Other studies have also shown that the dichotomous effect of MSCs was dependent on MSC:lymphocyte ratio where the homeostatic IL-2, IL-6 and IL-7 cytokines appeared to be involved^32,34^. Supporting this, we observed a stimulatory effect at low number of both nsMSCs and sMSCs on the proliferation of T cells isolated from the three groups compared to the effect of T cells alone. Indicating that even nsMSCs has the ability to stimulate the CD4+ T cells. For the Healthy and SLE group a reduced proliferation of CD4+ T cells in culture with nsMSCs were observed compared to sMSCs. This was not detected for the LN group and may indicate a different composition of lymphocyte subsets and activation levels of T cells in patients with LN, compared to SLE patients and healthy donors^3,46–48^. Surprisingly we were able to culture T cells alone for 10 days in absence of IL-2. There were no significant difference in the live cell population in cultures of T cell alone compared to T cells in co-culture with nsMSCs and sMSCs. Indicating a specific proliferation of unstimulated T cells when co-cultured with sMSCs. Najar *et al*. proposed that MSC-mediated inhibition was optimal at a 1:4 and 1:8 MSC:T cell ratio^32^, whereas others have reported inhibition at a 1:1 ratio^34^. Interestingly, nsMSCs increased the CD4+ T cells proliferation from healthy donors largely in the transwell system, indicating a partial contact dependent inhibitory effect of nsMCSs on CD4+ T cells proliferation, which we did not observe in sMSCs. nsMSCs expressed IL-7, IL-1β, and CXCL12, which their role in inducing proliferative response in T cells have been discussed^49–51^.

On the other side, several studies have shown that the activation of MSCs with proinflammatory cytokines can induce MSCs to be more immunosuppressive vs, nonstimulated MSCs^52–54^. Han *et al*. demonstrated that IFNγ and TNFα stimulated MSCs had an immunosuppressive effect *in vivo* in a tumor inflammatory environment and thereby promoted tumor growth. In a study by Sivanathan *et al*., IL-17 stimulated MSCs were more immunosuppressive than IFNγ stimulated or unstimulated MSCs. IFNγ together with TNFα, IL-1α, or IL-1β induced the immunosuppressive function of MSCs by upregulating inducible nitric oxide synthase *(iNOS)* and many chemokines attracting immune cells^52^. Ren *et al*. showed that only the combination of all cytokines gave the highest immunosuppressive response of MSCs. In addition, the maximum inhibition was observed on activated T cells^52^. In our experiments, we focused on the CD4^+^ T cells and the effect of sMSCs and usMSCs on the proliferation and differentiation of these. Several studies and review paper have addresses the dual function of immunosuppressive vs immunestimulating potential of MSCs^22,55^. The MSCs may have different properties and functions depending on the inflammatory milieu and disease setting. In the developing TLS, the recruitment of naïve T cells by activated MSCs may be important for the expansion and activation of tissue specific effector T cells and need to be further investigated.

Our results suggest that an inflammatory environment could promote MSCs to secrete cytokines such as IL-1β and TNF-α, in addition to CCL19 to recruit T cells at the site of inflammation. It is known that IL-17 induces MSCs production of CXCL13 to recruit B cells, and that Th2-type cytokines and IL-23 induce naïve B cell proliferation and differentiation into plasma cells at a site of TLS formation^10,28,56,57^. We also showed that MSCs express ICAM1, VCAM1, and HLA-DRA, and that the interaction with T cells resulted in activation of CD4+ T cells and differentiation into inflammatory T helper cells like Th2 and Th17. The dual impact of MSCs in suppressing and inducing Th1, Th2, and Th17 response has been extensively discussed^58–62^. It has been shown that CD4+ T cells in LN patients are stimulated and activated towards a Th1, Th2, and Th17 orientation^2,47,63,64^, and the involvement of IL-1β, IL-6, IL-21, IL-23, and HLA-DR play pivotal roles in Th2 and Th17 responses^16,65–68^. This could partially explain our results, which indicate that high expression levels of these cytokines in sMCSs co-cultured with T cells isolated from healthy donors at high ratio potentially promote both Th2 and Th17 response at the same time. These findings, to the best of our knowledge, have previously not been reported. However, T cells from SLE and LN patients did not significantly differentiate into the different Th subsets. There are several possibilities for this observation. First, if the patients were in an active state of the disease the response might have been stronger. Second, the already activated T cells from SLE and LN patients were not able to differentiate further. Third, the activated T cells in SLE and LN patients were not within the circulation but located in effected tissues, and thus could not be isolated from blood.

Rangel-Moreno *et al*. demonstrated that Th17, IL-17-producing CD4+ T cells, can induce TLS formation in the lung of mouse model lacking LTi cells^57^. They explained that IL-17 could induce CCL19 and CXCL13 production in stromal cells, which lead to recruitment of B and T cells. In addition, IL-17 could also contribute to maintenance of the FDCs network^57^. Peters *et al*. showed that Th17 cells could promote TLS formation in the central nervous system in a mouse model of multiple sclerosis comprising T cells, plasma cells, and distinct B cell aggregation surrounded by reticulin as a lymphatic tissue supporting network^69^. The role of Th2 response/subtype/cells in TLS development remains elusive. Stromal precursor cells could propagate to mature FDCs upon LTβR activation by α1β2 integrin on T cells^70^. Nayar *et al*. demonstrated that type 2 (Th2) cytokines, IL-4 and IL-13, could trigger the transition of residential stromal cells^71^ and adipose-derived stem cells^72^ to “lymphoid” stromal cells. Type 2 cytokine (IL-4) produced by Th2 cells could also induce expression of LTα1β2 on naive T cells required for future CD4+ T cells (LTi) and MSCs (LTo) interactions^73^. These facts are emphasizing the critical roles of Th2 and Th17 cells in promoting local inflammation and TLS development.

In the present study, we demonstrate that the expression of proinflammatory cytokines IL-1β and TNF-α, in addition to ICAM1, VCAM1, HLA-DR, and CCL19 were induced in sMSCs compared to non-stimulated cells. However, the expression of B cell homing cytokine CXCL13 and IL-23 could not be induced by single cytokine stimulation. Contrary, the sMSCs in co-cultured with T cells isolated from the healthy donors, expressed high levels of CXCL13, IDO, HLA-DR, IL-21, and IL-23, but not CCL19. Interestingly, this expression pattern was not observed in sMSCs co-cultured with T cells from SLE and LN patients. These patients were pre-treated with acetylsalicylic acid, hydroxychloroquine, methotrexate, and mycophenolate mofetil (MMF), which are known to affect the immune system and thereby effecting cytokine production, T cell activation and proliferation^74,75^. Hence, this could indirectly effect the response and capacity of the MSCs. However, our findings indicate that MSCs in an inflammatory environment may be important in both attracting T cells and B cells, in addition to contributing to the inflammatory response at an early phase. Our data suggest that MSCs after *in vitro* stimulation might acquire FRC-like phenotype expressing CCL19. However, after co-culturing with T cells they acquired an FDC-like phenotype expressing CXCL13 to attract B cell to the site of inflammation. This is in accordance with the findings of Peduto *et al*.^38^. They suggested that gp38+ “lymphoid” stromal cells could consist of different stromal cell subsets or maturation stages based on genes and markers profile. They identified a CXCL13 expressing gp38+ stromal cells subset distinct from the CCL19 expressing subset^38^. It has been discussed that most of FDCs rise from migratory precursors with a mesenchymal origin^76^. Muñoz-Fernández *et al*. proved that FDCs originate from bone marrow stromal cell progenitors^77^. It has been shown that stimulation of FDC stromal precursor cells through LTβR and TNFR1 drives their differentiation to FDCs^70,78^.

The development of TLS within chronic inflamed tissues has been extensively reviewed^79,80^. Formation of TLS within target organs in autoimmune diseases like rheumatoid arthritis, Sjögren syndrome, and SLE, among others, indicate a tissue specific development of these structures^79^. TLS has been associated with the severity of autoimmune diseases and there are increasing evidence that tissue specific stromal cells play a role in the induction of TLS^12^.

As a new model for the formation of kidney-specific TLS and progression of LN in lupus-prone mice we propose that migratory or residential MSCs in the pelvic wall within the kidney of lupus-prone mice could potentially reprogram into LTo cells upon stimulation at an early stage of SLE progression. This might result in proinflammatory cytokine production and overexpression of adhesion molecules that can lead to activation and recruitment of residential CD4+ T cells as LTi cell and consequently LTo/LTi cells crosstalk. The LTo/LTi cells interaction could promote CD4+ T proliferation and differentiation toward Th2 and Th17 subsets, which continue as a positive loop and aberrant accumulation of other immune cells and enlargement of networks of lymphatic and stromal cells. All prior events lead to the development of large TLS with GCs reaction and local autoantibody production, which may result in the progression of LN. The role of autoantibody production in TLS need further studies.

## Methods

### Animals

(NZBxNZW)F1 (B/W) were obtained from Jackson Laboratory (Bar Harbor, Maine, USA). Treatment and care of animals were conducted in accordance with guidelines of the Norwegian Ethical and Welfare Board for Animal Research, and the Norwegian Food Safety Authority (FOTS) approved the study.

### Anti-dsDNA antibody enzyme-linked immunosorbent assay (ELISA) and measurement of proteinuria

The level of anti-dsDNA antibody in lupus-prone mice serum was measured as described previously^81^. Blood samples were taken every week until positive ELISA test, and thereafter every second week until proteinuria was detected in urine. The protein in the urine was determined by urine dipstick (Bayer Diagnostics, Bridgend, United Kingdom): 0–1+ (<1 g/liter), was regarded as physiological proteinuria; 2+ (≥1 g/liter to 3 g/liter) and 3+ (≥3 g/liter to 20 g/liter) and 4+ (≥20 g/liter). Proteinuria was defined as 4+.

### Antibodies

CD4-VioGreen (130-106-655, Miltenyi Biotec), Anti-CCR10-PE (clone: REA326, 130-104-822, Miltenyi Biotec), CD183 (CXCR3)-PE-Vio770 (clone: REA326, 130-104-822, Miltenyi Biotec), CD194 (CCR4)-APC (clone: REA279, 130-103-813, Miltenyi Biotec), CD196 (CCR6)- PE-Vivo-615 (clone: REA190, 130-107-142, Miltenyi Biotec), Propidium iodide (130-093-233, Miltenyi Biotec), NESTIN rabbit-anti-mouse (ab7659, Abcam), SCA1 rat-anti-mouse (ab25195, Abcam), PDGFRα goat-anti-mouse (AF1062, R&D Systems), Alexa Flour 405 donkey-anti-Rabbit (ab175651, Abcam), Alexa Flour 488 donkey-anti-goat (A-11055, Thermo Fisher Scientific), Alexa Flour 594 donkey-anti-rat (A-21209, Thermo Fisher Scientific), PerCP-CyTM 5.5 CD45.2 conjugated mouse-anti-mouse (552950, BD Biosciences), ICAM1 rabbit-anti-human (ab53013, Abcam), VCAM1 rabbit-anti-human (ab134047, Abcam), IL-1β rabbit-anti-human (ab2105, Abcam), TNF-α rabbit-anti-human (ab9635, Abcam), actin rabbit-anti-human (A2066, Sigma-Aldrich), rabbit anti goat (81–1620, Invitrogen), goat anti rabbit (65–6120,Invitrogen), CD3 rabbit-anti mouse (AD452, Dako), and B220 rat-anti-mouse (MAB1217, R&D Systems).

### Immunofluorescent staining and Fluorescence Confocal Microscopy

Detection of NESTIN, SCA1, PDGFRα, and CD45 were performed on kidney cryosections (4 µm) from (NZBxNZW)F1 mice. Dried sections were fixed in 4% paraformaldehyde for 5 minutes and washed twice in 1xPBS for 5 minutes. Sections were blocked with 10% donkey serum in PBS for 30 minutes, and incubated for 30 minutes with primary antibodies (NESTIN 10 μg/ml, Sca1 10 μg/ml, and PDGFRα 2 μg/ml). Washed sections were incubated with secondary antibodies Alexa Flour 405, Alexa Flour 488, Alexa Flour 594, Alexa Flour 647 (all three 2 μg/ml) and PerCP-CyTM 5.5 mouse-anti-mouse CD45.2 conjugated (4 μg/ml). Slides were washed, dried, and mounted using Mowiol (Sigma-Aldrich). The sections were examined by LSM780 AxioObserver (Carl Zeiss, Oberkochen, Germany), and the intensity were measured by drawing a line in the area of interest. The intensity of different signals (number based) for the whole length of the line were determined. For each image, two lines were drawn, one in the CD45+ area and one in PDGFRα+ area.

### Immunohistochemistry

Detection of CD3 positive T cells (Dako, 3 μg/ml) and B220 positive B cells (R&D systems, 0.83 μg/ml) in 4 µm paraffin embedded kidney sections were performed as described previously^36^. Envision+ system-HRP/DAB anti rabbit detection kit (K4011, Dako) and Polink-2 Plus HRP Anti-Rat DAB detection Kit (D46-15, Golden Bridge International) were used to detect CD3 and B220 respectively.

### RNA isolation and qPCR of murine kidneys

Total RNA from kidney tissue (10 mg) and dissected TLS and kidney tissue preserved in RNAlater (Ambion Inc., Texas) was isolated with the EZ1 RNA Tissue Mini Kit (Qiagen, Nordic, Norway) according to the manufacturers’ protocol. The isolated RNA was then reverse-transcribed with random primers using High-Capacity cDNA Reverse Transcription kit (4368814, ThermoFisher Scentific). Real-time PCR was performed on an ABI Prism 7900HT Sequence Detection System (Applied Biosystems) with the following TaqMan gene expression assays: TBP, LTB, LTBR, CCL19, CXCL13, VCAM1 and ICAM1 (Supplementary Table S4). TaqMan Fast Universal PCR master mix (2X) and gene expression assays were all obtained from Applied Biosystems (Foster City, USA). Gene expression was normalized against the reference gene TBP for each mouse. Average of young mice (4 w.o, n = 3) served as reference for total kidney and dissected TLS and kidney tissue and changes in gene expression were calculated with the ΔΔCT method shown as fold change (FC). Young mice age 4–18 wo (n = 35), Ab+ mice age 18–28 wo (n = 28), and proteinuric mice 23–39 wo (n = 27).

### Healthy and SLE subjects

Peripheral blood samples were obtained from patients diagnosed with SLE and LN according to the 1997 American College of Rheumatology Revised Criteria for classification of SLE^82,83^. Peripheral blood samples from random healthy donors were obtained from the blood bank at University Hospital of North Norway (UNN, Tromsø Norway).

### MSCs, HMLE and HUV-EC-C stimulations

#### Single cytokine stimulations

Bone marrow-derived MSCs (Normal, Human; ATCC® PCS-500-012) (p.9) were plated in a flat-bottomed 6 well plate (Corning Inc, US) at 2.0 × 10^5^ cells/ml for 24 hours, following serum reduction for 18 hours in complete medium (ATCC PCS-500-030)(Supplementary Table S5) containing 3% fetal bovine serum (FBS). Cells were stimulated with the following cytokines IL-1β (201-LB, R&D Systems), TNF-α (210-TA, R&D systems), IFN-α (11100-1, R&D systems), INF-γ (285-IF, R&D systems), IL-6 (206-IL, R&D systems), and IL-10 (217-IL, R&D systems) at 2, 4, 8, 16, 20, 25, and 35 ng/ml for 24 hours in complete medium containing 0.1% FBS.

#### Combined cytokine stimulations

MSCs (p.9) were plated in 12 well plates at 1.0 × 10^5^ cells/ml for 24 hours, and starved in complete medium containing 3% FBS for 18 hours. Based on the results from the single cytokine stimulation, cells were stimulated with IL-1β (8 ng/ml) and TNF-α (8 ng/ml) in complete medium containing 0.1% FBS for 1–72 hours. The same experiment was performed with the HUV-EC-C cells (Normal, Human; ATCC® CRL-1730TM) and HMLE cells (Robert Weinberg, Whitehead Institute for Biomedical Research and Department of Biology, Massachusetts Institute of Technology). HMLE cells were not starved prior to stimulation (Supplementary Table S3).

### T Cell Isolation

Lymphoprep^TM^ (STEMCELL Technologies) was used to isolate peripheral blood mononuclear cells (PBMCs) from buffy coat or heparinized blood obtained from the blood bank (UNN, Tromsø Norway), according to the manufacturer’s instructions. Enriched CD4+ T cells were obtained by negative selection from lymphoprep-PBMCs pellets using the magnetic CD4+ T cell human isolation kit (130-096-533 Miltenyi Biotec) and LS Column (Miltenyi Biotec).

### T cell co-cultures

#### Direct contact

Bone marrow-derived MSCs were cultured in complete medium at 37 °C in 5% CO_2_. Cells (p.9) were plated in a flat-bottomed 24 well (Corning Inc, US) for proliferation assay (2.0 × 10^3^) and flow cytometry analysis (5.0 × 10^3^) for 24 hours. Cells were starved for 18 hours in complete medium containing 3% FBS, stimulated with 8 ng/ml IL-1β and TNF-α in complete medium containing 0.1% FBS for 6 hours, washed twice with 1xPBS. CD4+ T cells were subsequently added to the wells at 1:1 and 1:100 ratios (MSCs: CD4+ T cells). The same experiment was performed with the HUV-EC-C cell line.

#### Transwell system

MSCs and HUV-EC-C cells were plated in a 24 well cell culture insert companion plate Falcon^®^ (734-0067, Corning) at 2.0 × 10^3^ cell/ml. CD4+ T cells were subsequently added to the PET membrane inserts Falcon^®^ with 0.4 μm pore size (734-0036, Corning) at 1:1 and 1:100 ratios for use in the proliferation assay. MSCs and HUV-EC-C cells were plated in a 24 mm Transwell^®^ plate at 8.0 × 10^3^ cell/ml and CD4+ T cells were added to polyester membrane insert with 3 µm pore size (CORN3452, Corning) at 1:1 and 1:100 ratios for use in flow cytometry analysis.

### Proliferation Assay

At day 0, 5, 7, and 10, the proliferation of CD4+ T cells co-culture with sMSCs, nsMSCs (direct contact and Transwell system), sHUV-EC-C cells, nsHUV-EC-C cells (direct contact) and CD4+ T cells cultured alone were measured. Cell suspensions of CD4+ T cells were transferred to new wells and added 50 μL of AlamarBlue® (DAL1100, ThermoFisher Scientific) to a final concentration of 10% (V/V). After 3 hours of incubation at 37 °C in 5% CO_2_, fluorescence of reduced AlamarBlue® in the supernatant was measured (λ_ex_ = 540 nm, λ_em_ = 590 nm) with a CLARIOstar® microplate reader (BMG LABTECHq).

### RNA isolation and qPCR of cells

Cells were harvested and total RNA was isolated using TRIzol (ThermoFisher Scientific), and High Capacity cDNA Reverse Transcription Kit (Applied Biosystems) was used to synthesize cDNA. Real-time qPCR was performed with the LightCycler® Analysing machine (Roche Holding AG) using TaqMan gene expression assays (ThermoFisher Scientific) (Supplementary Table S4) and TaqMan® Fast Universal Master Mix (Applied Biosystems). Total cDNA input was normalized to the reference gene measured in parallel qPCR reactions. Real-time qPCR data is presented as relative expression using ΔΔCT method in which relative expression = 2^−ΔΔCT^. Each sample was run in duplicates and threshold cycle (C_t_) values were averaged from each reaction. TBP was used as reference gene. Where no Ct values were detected for the controls the Ct value were set to 37 for statistical calculations.

### FACS

Surface marker staining was performed on enriched CD4+ T cells from PBMCs at day zero, CD4+ T cells co-culture with sMSCs, nsMSCs (direct contact and Transwell system), sHUV-EC-C cells, nsHUV-EC-C cells (direct contact and Transwell system) and CD4+ T cells cultured alone at day ten. Cells were pelleted down and resuspended in 100 μl staining buffer (containing phosphate-buffered saline (PBS), pH 7.2, 0.5% bovine serum albumin (BSA), and 2 mM EDTA). Antibodies CD4-VioGreen (0.034 μg/ml), CCR10-PE (0.034 μg/ml), CD183 (CXCR3)-PE-Vio770 (0.02 μg/ml), CD194 (CCR4)-APC (0.068 μg/ml), CD196 (CCR6)-PE-Vivo-615 (0.013 μg/ml) were added and incubated for 10 min at 4 °C in the dark. Cells were washed with 1 ml staining buffer, pelleted and resuspended in 100 μL MACSQuant running buffer (Miltenyi Biotec). 5 μL of Propidium iodide (Miltenyi Biotec) was added shortly before running the samples on FACSARIA III (BD Biosciences). Data were analyzed with FlowJo software (v10, FlowJo LLC). To compensate optimally for fluorescence spillover from fluorochrome-conjugated antibodies, the MACS Comp Bead Kit, anti-human (130-104-187, Miltenyi Biotec) and anti-mouse (130-097-900, Miltenyi Biotec) Ig_κ_(Miltenyi Biotec) were used.

### Immunoblotting

Cells harvested with RIPA buffer containing phosphatase and protease inhibitors (Thermo Fisher Scientific) were sonicated (Bioruptor 300, Diagenode) for 5 minutes and protein concentration was measured using Direct Detect® Spectrometer (Merck Millipore). Boiled samples (20 μg protein, 4 μl LDS, and 1.4 μl reducing agent) were separated on Bis-Tris protein gel (Thermo Fisher Scientific) and transferred to nitrocellulose membrane (LI-COR Biosciences). The membrane was blocked with 5% nonfat skim milk (Bio-Rad) for one hour and incubated with primary antibodies (ICAM1 0.03 μg/ml, VCAM1 0.22 μg/ml, IL-1β 2 μg/ml and, TNF-α 2 μg/ml) overnight at 4 °C. Proteins were detected using appropriate HRP-conjugated antibodies (rabbit anti goat 0.75 μg/ml, goat anti rabbit 0.67 μg/ml) and SuperSignal West Pico chemiluminescent substrate (Thermo Fisher Scientific) to obtain images with ImageQuant LAS 4000 Imager (GE Healthcare Life Sciences).

### Statistics

We performed Two-way ANOVA with Post-hoc analysis (Sidak’s and Dunnett’s multiple comparisons test) for cell culture qPCR data, Two-way ANOVA with Post-hoc analyses (Dunnett’s and Tukey’s multiple comparisons tests) for proliferation data, One-way ANOVA with Post-hoc analysis (Sidak’s multiple comparisons test) for Flow cytometry data, and Kruskal-Wallis test with Dunn’s Multiple Comparison Test for kidney tissue qPCR data. A *p*-value of ≤0.05 was considered statistically significant and data are presented as mean ± SEM.

### Study approval

For animal studies, the Animal Welfare Board, at the UiT–The Arctic University of Norway and the Norwegian Food Safety Authority (NFSA, Mattilsynet) approved all procedures. Patients and healthy individuals were recruited through the Department of Rheumatology, University Hospital of North Norway and the Regional Committees for Medical and Health Research Ethics (REK) approved the project. Informed consent was obtained from all patients and healthy individuals before sample collection. The REK approved all experimental protocols involving human participants and samples (REK 2015/1315). We confirm that all experiments were performed in accordance with relevant guidelines and regulations.

## Electronic supplementary material

Supplementary Files
