# Supplementary material for: Mesenchymal stem cells and T cells in the formation of Tertiary Lymphoid Structures in Lupus Nephritis
Source: Sci Rep. 2018 May 18;8:7861. doi: 10.1038/s41598-018-26265-z (PMC5959845; doi:10.1038/s41598-018-26265-z)
Supplement: Supplementary file 1 — Supplementary Files [file 41598_2018_26265_MOESM1_ESM.pdf]

TITLE PAGE

Mesenchymal stem cells and T cells in the formation of Tertiary Lymphoid Structures in Lupus Nephritis

Running title: Stromal cells in tertiary lymphoid structures

S. Esmaeil Dorraji[1], Aud-Malin K. Hovd[1], Premasany Kanapathippillai[1], Gunnstein Bakland[2,3], Gro Østli Eilertsen[2,3], Stine L. Figenschau[1], Kristin A. Fenton[1]

[1]RNA and Molecular Pathology Research Group, Institute of Medical Biology, Faculty of Health Sciences, UiT, The Arctic University of Norway, Tromsø, Norway; [2]University Hospital of Northern Norway, Tromsø, Norway; [3]Molecular Inflammatory Research Group, Institute of Clinical Medicine, Faculty of Health Sciences, UiT, The Arctic University of Norway, Tromsø, Norway

Correspondence: Kristin A. Fenton RNA and Molecular Pathology Research Group, Institute of Medical Biology, Faculty of Health Sciences, UiT, The Arctic University of Norway, MH-building, 9037 Tromsø, Norway. Phone: +4777664834. E-mail: kristin.fenton@uit.no

Supplementary figures

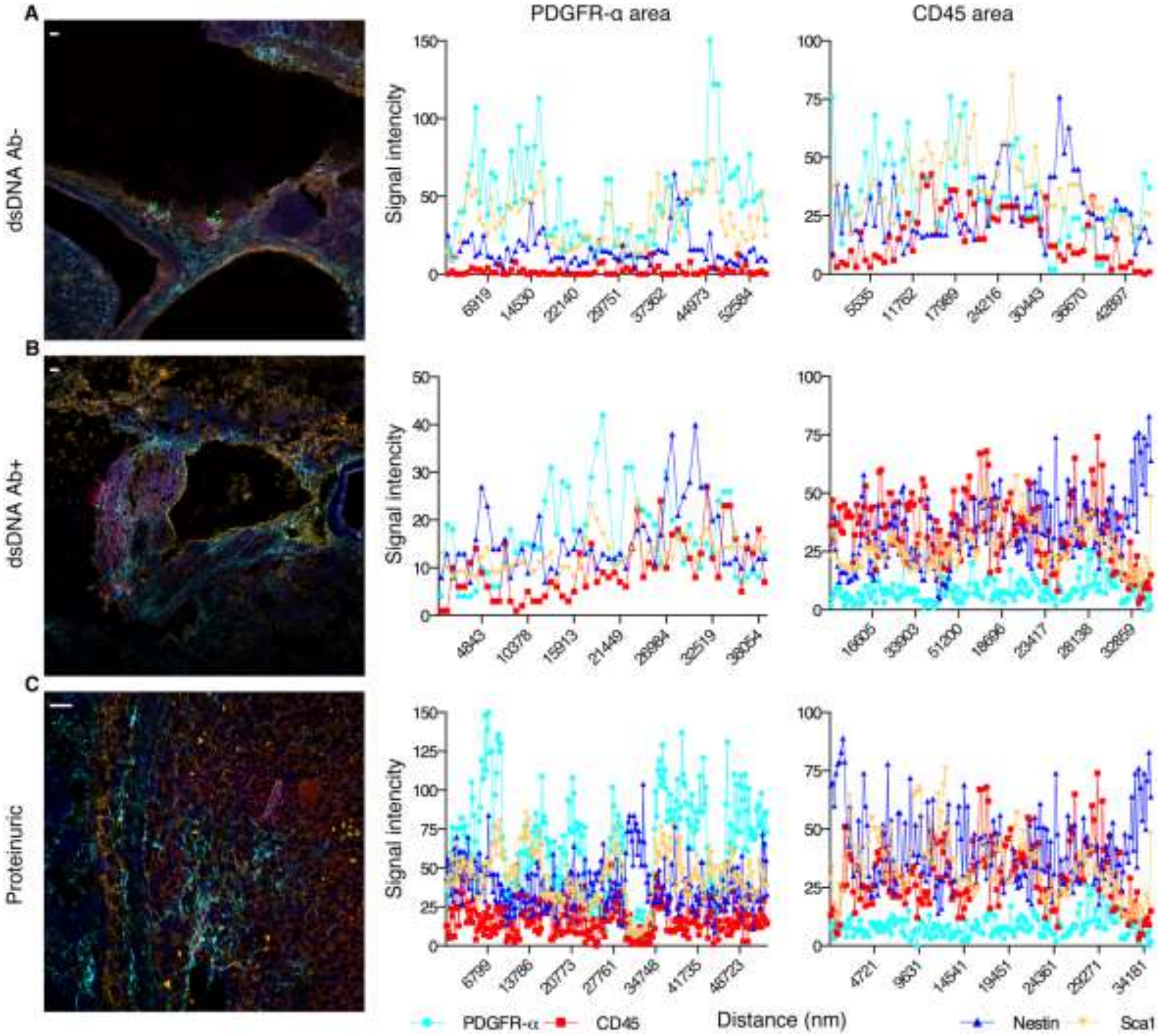

Supplementary Figure S1: Nestin, Sca1, PDGFRα, and CD45 Signal intensity. The signal intensity graphs represent the intensity of four different signals Nestin, Sca1, PDGFRα, and CD45. Each group (**A**) Ab-, (**B)** Ab+, and (**C**) proteinuric contain two graphs, which indicate PDGFRα positive (CD45 negative) area and CD45 positive (PDGFRα negative) area.

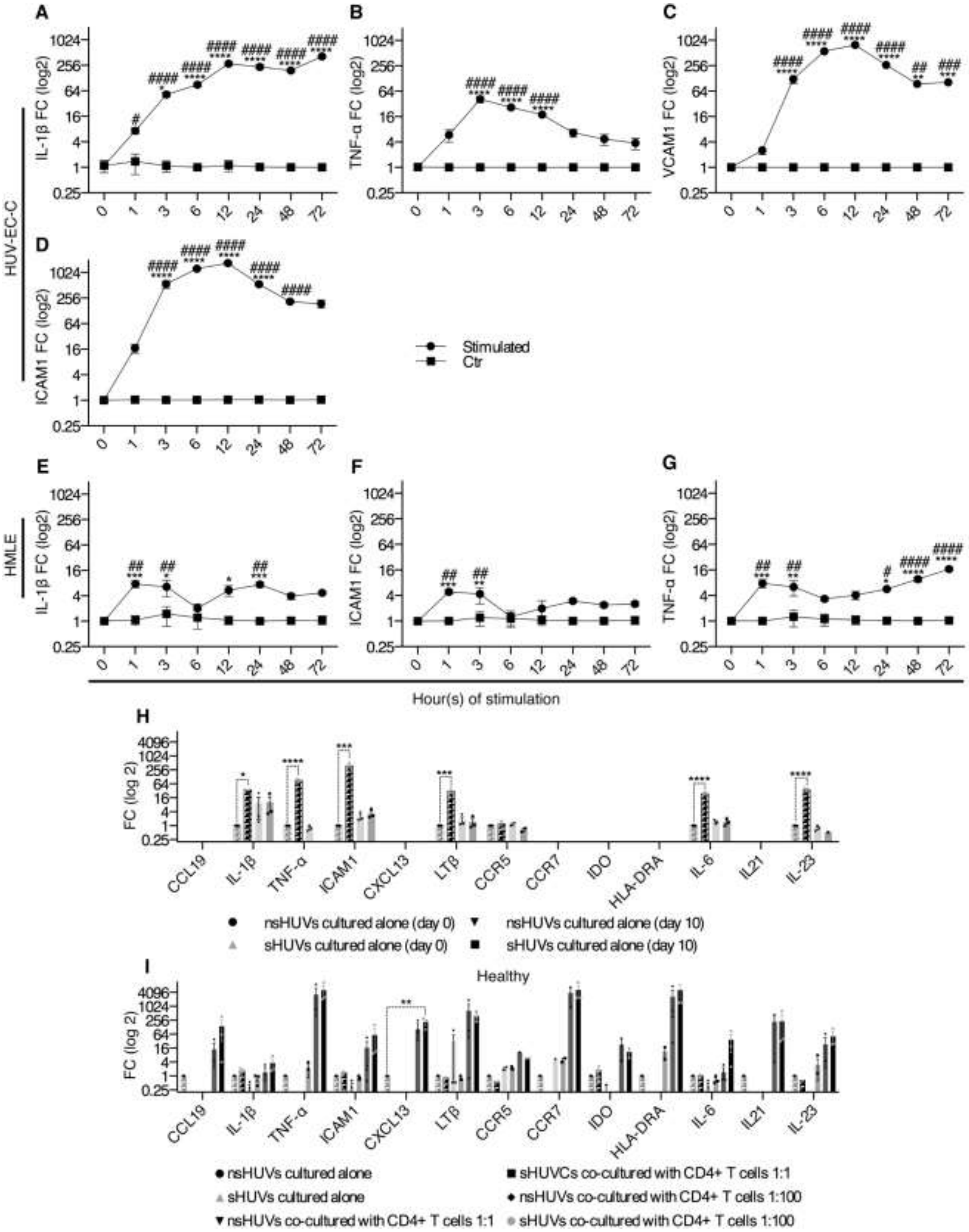

Supplementary Figure S2: Stimulated HMLE and HUV-EC-C cells express proinflammatory cytokines and adhesion proteins. HUV (n=3) cells were stimulated with IL-1β and TNF-α (8

ng/ml) and mRNA expression of (**A**) IL-1β, (**B**) TNF-α, (**C**) VCAM1, and (**D**) ICAM1 were measured after 1-72h. HMLE (n=3) cells were stimulated with IL-1β and TNF-α (8 ng/ml) and mRNA expression of (**E**) IL-1β, (**F**) ICAM1, and (**G**) TNF-α were measured after 1-72h. Results are shown as FC mean ± SEM compared to Ctr at the same time points. (**H**) HUV (n=3) were stimulated for 6h with IL-1β and TNF-α (8 ng/ml), and analyzed for the mRNA expression of CCL19, IL-1β, TNF-α, ICAM1, CXCL13, LTβ, CCR5, CCR7, IDO, HLA-DRA, IL-6, IL-21 and IL-23 at day 0 and day 10. Results are given as FC mean ± SEM compared to nsHUV at day 0. (**I**) nsHUV and sHUV in co-culture with CD4+ T cells at 1:100 ratio from Healthy donors (n=3). Results are given as FC mean ± SEM compared to nsHUV at day 10. **(A-G**) Two-way ANOVA with Post-hoc analysis Sidak's (*) and Dunnett's (compare within a group toward zero hour time point, marked with #) multiple comparisons tests. */#P < 0.05; **/##P < 0.01; ***/###P < 0.001; ****/####P < 0.0001.

(**H-I**) One-way ANOVA with Post-hoc analysis Dunnett's multiple comparisons test (compared within a group toward nsMSCs or nsHUV at given time point, marked with *). *P < 0.05; **P < 0.01; ***P < 0.001; ****P < 0.0001.

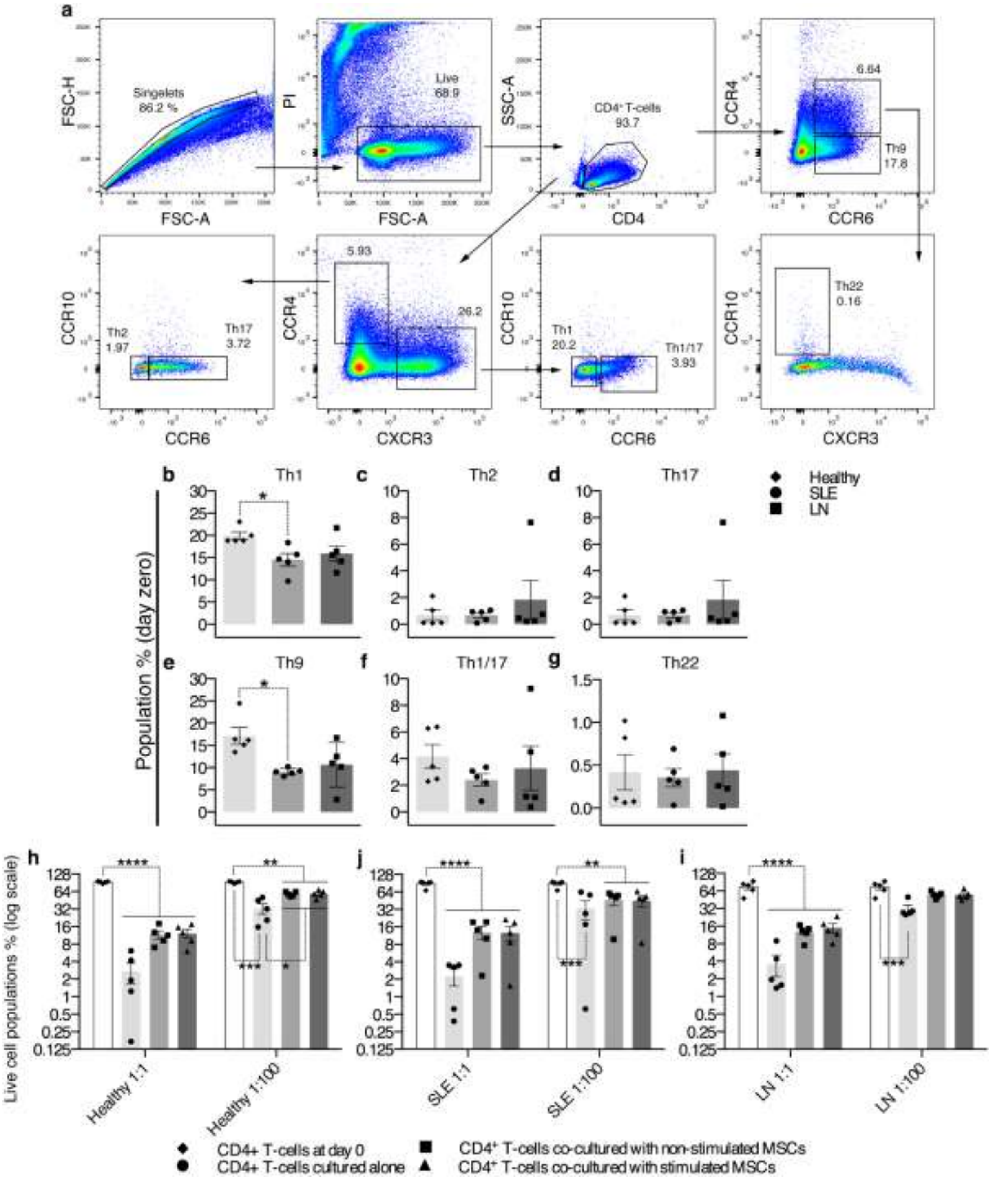

Supplementary Figure S3: Higher Th1 and Th9 subsets were observed in T cells from Healthy group compared to SLE and LN.

(**A**) Illustrated gating strategy used to define the different Th cells subsets. Purified CD4+ T cells from the Healthy donors (n=5), SLE patients (n=5), and LN patients (n=5) were analyzed by FACS. After gating for singlets and subsequently live cells; cells were analyzed for CD4. The CD4+ cells were used further to analyze Th cells subsets based on expression of surface markers: (**B**) CXCR3+CCR10-CCR6- for Th1 (**C**) CCR4+CXCR3-CCR10-CCR6- for Th2, (**D**)

CCR4+CXCR3-CCR10-CCR6+ for Th17, (**E**) CCR4-CCR6+ for Th9, (**F**) CCR4-CXCR3+CCR6+ for Th1/17cells, and (**G**) CCR4+CCR6+CCR10+ for Th22. After co-culturing at 1:1 ratio and 1:100 ratio with nsMSCs and sMSCs or cultured alone, CD4+ T cells from Healthy, SLE and LN groups were analyzed by FACS. The % of live T cell population from (**H**) Healthy (n=5), (**J**) SLE (n=5), and (**I**) LN (n=5) group were measured at day zero and after 10 days in co-culture. Data are given as % cell population mean ± SEM. One-way ANOVA with Post-hoc analysis Sidak's multiple comparisons test (compare within groups, marked with *). *P < 0.05; **P < 0.01; ***P < 0.001; ****P < 0.0001.

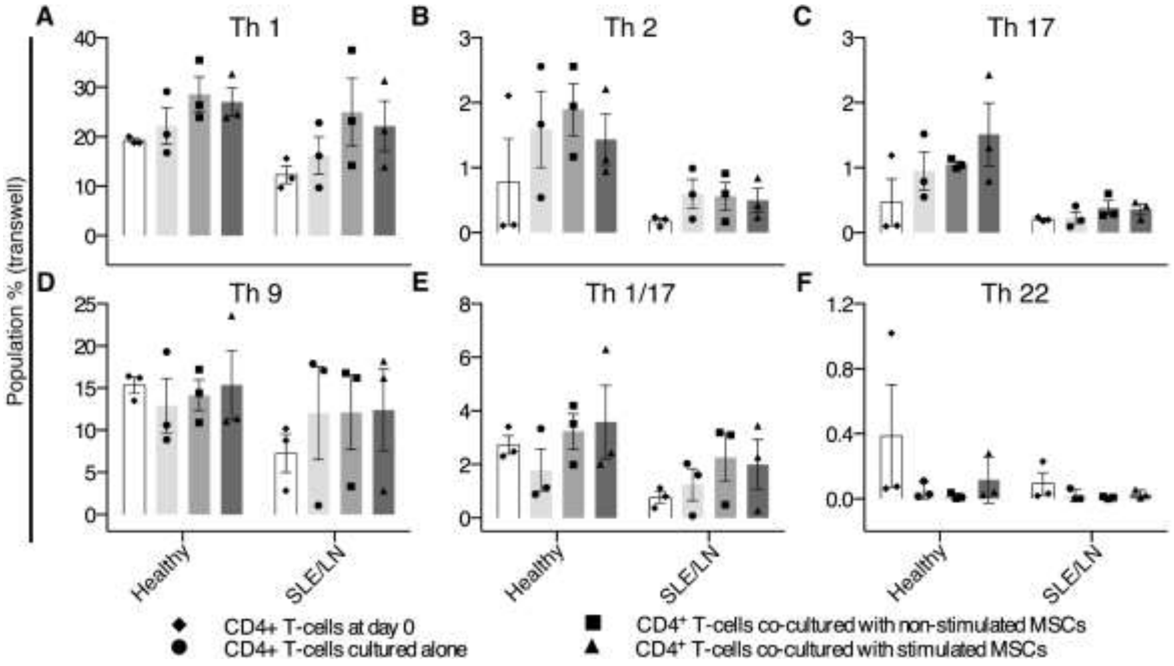

Supplementary Figure S4: CD4+ T cells from the Healthy group do not differentiate into Th2 and Th17 subsets in indirect co-culture with sMSCs.

To analyze if the differentiation was contact dependent CD4+ T cells from the Healthy (n=3) or Lupus (n=3, SLE n=1, LN n=2) groups were co-cultured at 1:100 ratio (MSCs:CD4+ T cells) with nsMSCs, sMSCs or cultures alone in a Transwell system. The T cells were analyzed for (**A**) Th1, (**B**) Th2, (**C**) Th17, **(D**) Th9, (**E**) Th22, and (**F**) Th1/17 subtypes at day zero and at day 10. Data are shown as % population mean ± SEM. One-way ANOVA with Post-hoc analysis Sidak's multiple comparisons test (compare within groups, marked with *). *P < 0.05.

Supplementary Tables

Supplementary Table S1: Gene expression in MSCs after single stimulation with TNF-α and IL-1β.

			Stimulated with IL-1β								Stimulated with TNF-α							
			Ctr	2ng	4ng	8ng	16ng	20ng	25ng	35ng	Ctr	2ng	4ng	8ng	16ng	20ng	25ng	35ng
TBP	Ct value	Avg	25.84	25.72	25.79	25.73	25.48	25.50	25.58	25.95	24.96	25.15	25.23	25.26	25.14	24.95	24.99	25.28
		SD	0.02	0.06	0.19	0.11	0.14	0.05	0.29	0.10	0.14	0.19	0.16	0.17	0.14	0.21	0.07	0.17
CCL21	Ct value	Avg	35.46	35.64	36.09	35.52	35.16	35.86	35.53	35.99	36.85	36.11	36.30	36.82	36.98	37.02	35.91	36.30
		SD	0.98	0.40	0.23	0.79	0.35	0.06	0.45	0.07	0.65	0.27	0.69	0.35	0.92	0.93	0.59	0.59
	Fold change	Avg	1.11	0.84	0.64	1.00	0.99	0.58	0.78	0.73	1.04	1.77	1.73	1.21	0.99	0.82	1.89	1.68
		SD	0.69	0.27	0.22	0.58	0.24	0.03	0.19	0.04	0.34	0.53	0.67	0.44	0.54	0.35	0.56	0.57
LTβr	Ct value	Avg	23.36	23.43	23.96	23.55	23.73	23.91	23.99	24.26	23.53	23.70	23.85	23.88	23.86	23.95	24.11	24.15
		SD	0.21	0.13	0.22	0.04	0.09	0.22	0.12	0.07	0.08	0.08	0.12	0.11	0.11	0.16	0.20	0.20
	Fold change	Avg	1.00	0.89	0.63	0.82	0.61	0.53	0.52	0.57	1.01	0.92	0.89	0.89	0.77	0.64	0.64	0.72
		SD	0.14	0.05	0.03	0.03	0.03	0.08	0.04	0.03	0.14	0.13	0.10	0.10	0.09	0.08	0.04	0.07
CXCL13	Ct value	Avg	ND	ND	ND	ND	ND	ND	ND	ND	ND	ND	ND	ND	ND	ND	ND	ND
		SD	ND	ND	ND	ND	ND	ND	ND	ND	ND	ND	ND	ND	ND	ND	ND	ND
	Fold change	Avg	ND	ND	ND	ND	ND	ND	ND	ND	ND	ND	ND	ND	ND	ND	ND	ND
		SD	ND	ND	ND	ND	ND	ND	ND	ND	ND	ND	ND	ND	ND	ND	ND	ND
CXCL12	Ct value	Avg	22.99	23.02	23.56	23.09	22.91	22.93	23.12	23.68	23.11	23.93	23.94	24.04	24.06	23.96	24.14	24.33
		SD	0.09	0.03	0.19	0.03	0.18	0.17	0.08	0.12	0.04	0.11	0.08	0.16	0.11	0.22	0.09	0.17
	Fold change	Avg	1.00	0.91	0.64	0.88	0.84	0.80	0.74	0.65	1.00	0.59	0.62	0.61	0.50	0.47	0.47	0.47
		SD	0.08	0.05	0.02	0.04	0.13	0.12	0.06	0.06	0.10	0.05	0.02	0.12	0.01	0.03	0.04	0.04
RANKL	Ct value	Avg	ND	ND	ND	ND	ND	ND	ND	ND	ND	ND	ND	ND	ND	ND	ND	ND
		SD	ND	ND	ND	ND	ND	ND	ND	ND	ND	ND	ND	ND	ND	ND	ND	ND
	Fold change	Avg	ND	ND	ND	ND	ND	ND	ND	ND	ND	ND	ND	ND	ND	ND	ND	ND
		SD	ND	ND	ND	ND	ND	ND	ND	ND	ND	ND	ND	ND	ND	ND	ND	ND
PDPN	Ct value	Avg	28.14	27.08	27.42	26.95	27.47	26.90	27.59	28.27	PR	PR	PR	PR	PR	PR	PR	PR
		SD	0.21	0.38	0.72	0.23	0.66	0.37	0.82	0.47	PR	PR	PR	PR	PR	PR	PR	PR
	Fold change	Avg	0.95	1.96	1.63	2.17	1.36	1.82	1.25	0.99	PR	PR	PR	PR	PR	PR	PR	PR
		SD	0.08	0.51	0.52	0.50	0.72	0.36	0.51	0.32	PR	PR	PR	PR	PR	PR	PR	PR
CCR5	Ct value	Avg	34.09	34.61	34.36	34.68	34.22	34.21	34.59	34.60	34.08	33.22	32.83	34.11	34.86	35.10	34.95	35.05
		SD	0.08	0.49	0.88	0.84	0.34	0.20	0.29	0.22	2.29	0.29	0.28	0.93	0.98	0.60	0.97	0.83
	Fold change	Avg	1.00	0.66	0.89	0.69	0.74	0.70	0.59	0.75	1.73	1.92	2.62	1.35	0.64	0.44	0.60	0.64
		SD	0.04	0.19	0.47	0.33	0.22	0.09	0.21	0.12	1.58	0.50	0.31	1.09	0.34	0.17	0.40	0.40
CCR7	Ct value	Avg	35.82	34.46	34.81	34.76	34.59	34.54	35.09	35.04	36.11	37.38	37.87	37.50	37.00	36.92	37.70	37.52
		SD	0.71	0.34	0.59	0.47	0.39	0.44	0.28	0.49	0.30	0.63	0.55	0.39	0.30	0.21	0.71	0.60
	Fold change	Avg	1.06	2.43	1.96	2.02	1.87	1.93	1.35	1.87	1.00	0.45	0.35	0.44	0.53	0.48	0.34	0.44
		SD	0.49	0.63	0.51	0.61	0.39	0.67	0.22	0.61	0.12	0.17	0.17	0.08	0.14	0.01	0.16	0.21
IL-7	Ct value	Avg	ND	ND	ND	ND	ND	ND	ND	ND	ND	ND	ND	ND	ND	ND	ND	ND
		SD	ND	ND	ND	ND	ND	ND	ND	ND	ND	ND	ND	ND	ND	ND	ND	ND
	Fold change	Avg	ND	ND	ND	ND	ND	ND	ND	ND	ND	ND	ND	ND	ND	ND	ND	ND
		SD	ND	ND	ND	ND	ND	ND	ND	ND	ND	ND	ND	ND	ND	ND	ND	ND
IL-4	Ct value	Avg	29.57	29.45	29.57	29.52	29.28	29.61	29.29	30.02	PR	PR	PR	PR	PR	PR	PR	PR
		SD	0.22	0.05	0.34	0.09	0.05	0.12	0.16	0.00	PR	PR	PR	PR	PR	PR	PR	PR
	Fold change	Avg	1.00	1.01	0.96	0.97	0.96	0.75	0.98	0.77	PR	PR	PR	PR	PR	PR	PR	PR
		SD	0.14	0.07	0.15	0.11	0.06	0.10	0.06	0.01	PR	PR	PR	PR	PR	PR	PR	PR
VCAM1	Ct value	Avg	22.02	21.48	21.92	21.53	21.19	21.63	21.65	22.42	PR	PR	PR	PR	PR	PR	PR	PR
		SD	0.14	0.08	0.28	0.06	0.27	0.12	0.11	0.14	PR	PR	PR	PR	PR	PR	PR	PR
	Fold change	Avg	1.00	1.35	1.03	1.33	1.41	1.00	1.05	0.80	PR	PR	PR	PR	PR	PR	PR	PR
		SD	0.09	0.12	0.04	0.10	0.17	0.10	0.06	0.07	PR	PR	PR	PR	PR	PR	PR	PR

(Avg: average, ND: not detected, PR: presented in result section, SD: standard deviation).

Supplementary Table S2: Gene expression in MSCs after single stimulation with IL-10, IL-6, IFN- γ, and IFN-α.

		TBP		IL-1β				LTβr				CXCL13				CCLL19				VCAM1			
		Ct value		Ct value		Fold change		Ct value		Fold change		Ct value		Fold change		Ct value		Fold change		Ct value		Fold change	
		Avg	SD	Avg	SD	Avg	SD	Avg	SD	Avg	SD	Avg	SD	Avg	SD	Avg	SD	Avg	SD	Avg	SD	Avg	SD
Stimulated with IL-10	Ctr	25.65	0.37	27.80	0.92	1.21	0.96	21.84	0.00	1.02	0.26	ND	ND	ND	ND	ND	ND	ND	ND	23.92	0.19	1.21	0.96
	2ng	25.74	0.15	28.06	0.74	0.93	0.37	22.24	0.50	0.82	0.20	ND	ND	ND	ND	ND	ND	ND	ND	23.95	0.35	0.93	0.37
	4ng	25.60	0.20	26.46	0.46	2.26	0.79	21.83	0.08	0.89	0.05	ND	ND	ND	ND	ND	ND	ND	ND	26.46	0.64	2.53	0.89
	8ng	25.49	0.12	27.31	0.38	1.42	0.26	21.92	0.13	0.85	0.13	ND	ND	ND	ND	ND	ND	ND	ND	23.54	0.62	1.20	0.01
	16ng	25.69	0.18	27.13	1.09	2.81	2.00	22.27	0.35	0.77	0.10	ND	ND	ND	ND	ND	ND	ND	ND	26.46	0.07	26.46	2.33
	20ng	25.55	0.21	28.50	0.43	0.70	0.29	21.83	0.16	0.95	0.22	ND	ND	ND	ND	ND	ND	ND	ND	26.46	0.36	26.46	0.36
	25ng	25.34	0.17	27.32	0.57	1.28	0.51	21.82	0.06	0.82	0.06	ND	ND	ND	ND	ND	ND	ND	ND	26.46	0.15	26.46	0.69
	35ng	25.59	0.03	28.36	0.16	0.65	0.06	21.74	0.31	1.04	0.20	ND	ND	ND	ND	ND	ND	ND	ND	23.72	0.29	0.65	0.06
Stimulated with IL-6	Ctr	26.47	0.92	PR	PR	PR	PR	23.74	0.60	1.29	1.15	ND	ND	ND	ND	ND	ND	ND	ND	23.34	1.56	1.05	0.45
	2ng	25.62	0.09	PR	PR	PR	PR	23.27	0.18	0.76	0.06	ND	ND	ND	ND	ND	ND	ND	ND	21.95	0.19	1.45	0.18
	4ng	25.89	0.04	PR	PR	PR	PR	24.07	0.03	0.53	0.02	ND	ND	ND	ND	ND	ND	ND	ND	22.42	0.13	1.26	0.11
	8ng	25.76	0.02	PR	PR	PR	PR	23.72	0.25	0.62	0.10	ND	ND	ND	ND	ND	ND	ND	ND	22.46	0.11	1.13	0.07
	16ng	25.71	0.12	PR	PR	PR	PR	23.85	0.19	0.55	0.04	ND	ND	ND	ND	ND	ND	ND	ND	22.30	0.17	1.22	0.11
	20ng	25.75	0.10	PR	PR	PR	PR	24.19	0.21	0.45	0.04	ND	ND	ND	ND	ND	ND	ND	ND	22.27	0.08	1.28	0.08
	25ng	25.73	0.17	PR	PR	PR	PR	24.30	0.13	0.40	0.02	ND	ND	ND	ND	ND	ND	ND	ND	22.46	0.04	1.10	0.16
	35ng	25.91	0.21	PR	PR	PR	PR	24.36	0.16	0.44	0.02	ND	ND	ND	ND	ND	ND	ND	ND	22.84	0.46	0.96	0.17
Stimulated with IFN-γ	Ctr	26.00	0.30	30.02	0.79	0.95	0.14	23.68	0.51	1.03	0.09	ND	ND	ND	ND	ND	ND	ND	ND	PR	PR	PR	PR
	2ng	26.04	0.41	30.08	0.42	0.99	0.05	23.81	0.31	1.01	0.14	ND	ND	ND	ND	ND	ND	ND	ND	PR	PR	PR	PR
	4ng	26.19	0.40	30.81	0.44	0.66	0.03	23.81	0.25	1.13	0.15	ND	ND	ND	ND	ND	ND	ND	ND	PR	PR	PR	PR
	8ng	26.24	0.30	30.91	0.42	0.64	0.06	23.90	0.36	1.09	0.07	ND	ND	ND	ND	ND	ND	ND	ND	PR	PR	PR	PR
	16ng	26.36	0.19	31.28	0.32	0.56	0.20	24.29	0.28	0.91	0.13	ND	ND	ND	ND	ND	ND	ND	ND	PR	PR	PR	PR
	20ng	26.13	0.05	31.13	0.34	0.52	0.12	24.30	0.34	0.78	0.22	ND	ND	ND	ND	ND	ND	ND	ND	PR	PR	PR	PR
	25ng	26.35	0.30	30.60	0.34	0.90	0.36	24.59	0.32	0.72	0.04	ND	ND	ND	ND	ND	ND	ND	ND	PR	PR	PR	PR
	35ng	26.90	0.23	31.72	0.47	0.99	0.09	24.65	0.39	1.03	0.16	ND	ND	ND	ND	ND	ND	ND	ND	PR	PR	PR	PR
Stimulated with IFN-α	Ctr	26.25	0.35	PR	PR	PR	PR	23.85	0.62	1.01	0.18	ND	ND	ND	ND	ND	ND	ND	ND	PR	PR	PR	PR
	2ng	25.85	0.09	PR	PR	PR	PR	23.28	23.28	1.12	0.02	ND	ND	ND	ND	ND	ND	ND	ND	PR	PR	PR	PR
	4ng	25.80	0.10	PR	PR	PR	PR	23.48	23.48	0.98	0.11	ND	ND	ND	ND	ND	ND	ND	ND	PR	PR	PR	PR
	8ng	25.93	0.03	PR	PR	PR	PR	23.30	0.04	1.17	0.01	ND	ND	ND	ND	ND	ND	ND	ND	PR	PR	PR	PR
	16ng	25.84	0.17	PR	PR	PR	PR	25.66	0.76	0.24	0.14	ND	ND	ND	ND	ND	ND	ND	ND	PR	PR	PR	PR
	20ng	25.67	0.08	PR	PR	PR	PR	0.03	23.28	0.98	0.07	ND	ND	ND	ND	ND	ND	ND	ND	PR	PR	PR	PR
	25ng	25.80	0.25	PR	PR	PR	PR	0.08	23.28	1.32	0.42	ND	ND	ND	ND	ND	ND	ND	ND	PR	PR	PR	PR
	35ng	25.43	0.31	PR	PR	PR	PR	22.90	0.30	1.10	0.26	ND	ND	ND	ND	ND	ND	ND	ND	PR	PR	PR	PR

(Avg: average, ND: not detected, PR: presented in result section, SD: standard deviation).

Patient	Sex.	Age (y)	Disease duration	Diagnosis	Main clinical symptoms	Other clinical diagnosis	Immunological disruption	Nephritis stage/ duration	Disease activity (SLEDAI)	Current therapy
**1**	M	53	6 mth	SLE	Hem(Ly), Se (Pl), Diskoid ANA, Im	RP, Lymph	anti-dsDNA, LAC, low C3	-	4	MTX, PRED, HCQ
**2**	M	58	5 y	SLE	Rash, Ph, Hem-Le, Ar, ANA,	Sicca, pre-OP	SSA, SSB	-	3	HCQ, Ale, CF
**3**	F	36	17 y	SLE	Er, Rash, Hem-Le, Ar, ANA,	RP, SICCA	dsDNA, anti-sm, Comp, ACA-IgM, ACA-IgG, ACA-B2-IgA	-	7	ASA, AZA, HCQ, PRED, CF
**4**	F	20	12 y	SLE	Diskoid, rash, Ou, Ar, ANA, Im, Hem (Ly), CNS?	Alopecia	Anti-SSA	-	0	HCQ, AZA, LIT
**5**	F	82	42 y	SLE	Diskoid, Ar, CNS, Im, ANA	HypoT4, DM, Endo-PC, OP	Anti-SSA	-	0	AZA, PRED
**6**	F	33	6 y	LN	Rash, Ou, Ar, N, ANA, Im	RP	RNP, anti-dsDNA AB	Class 2 (6 y) Class 4 (3 y)	3	MMF, PRED, ENA, HCQ, CF
**7**	F	38	1 y	LN	N, ANA, Hem(Ly), Im,	CAC, IRI PCOS, APS, latent TB, SSS	ACA-B2-IgA, ACA-IgA, ACA-IgM, anti-RNP, anti-SSA, anti-SSB, anti-Sm	Class 3A (1 y) Class 5 (1 y)	0	MP, MMF, HCQ, ASA
**8**	F	73	1 y	LN	Ph, Rash, Hem(An), N, Im, Ar	TC (-89), PT, HBP,BCC-RE, Hip-OA, Epilepsy (-02)	Anti-Nucleus	Class 2 (6 mth)	1	Euro lupus protocol MP, CTX, PRED
**9**	F	64	28 y	LN	N, Im	APS	anti-DNA, , anti-smD, low C3,	Class 2 (16 y)	4	HCQ, PRED
**10**	F	61	31 y	LN	Ar, Discoid, N, Hem(Ly)	APS	dsDNA, SSA, low C3/C4 (normal at time of test)	Class 2B (16 y)	0	IVI, HCQ, PRED, MMF, BEL

Supplementary Table S4: Cell lines, media and growth kit:

Cell line	Basal media	Supplemented with
MSC (ATCC® PCS-500-012) *	Mesenchymal stem cell basal medium (PCS-500-030, ATCC)	Mesenchymal stem cell growth kit – for bone marrow derived MSCs (PCS-500-041, ATCC). Containing L-alanyl-L-Glutamine, FBS, rh GF-1, rhFGF-b and 1% penicillin-streptomycin (Sigma-Aldrich)
HMLE (Robert Weinberg, Whitehead Institute, MIT)	1:1 mixture of MEGM™ Mammary Epithelial Cell Growth Medium (Lonza) and DMEM/F12 (11320033, Thermo Fisher)	10ng/mL EGF 0.5 µg/mL hydrocortisone 0,01mg/mL insulin and 1% penicillin-streptomycin (Sigma-Aldrich)
HUV-EC-C (ATCC® CRL-1730TM)	F-12K Kaighn´s Modification of Ham´s F12 media (30-2004, ATTC)	0,1mg/mL of sodium heparin (H3393-199KU, Sigma Aldich), 0,03% endothelial cell growth supplement (ECGS, E2759, Sigma Aldrich), 1% penicillin-streptomycin (P0781-100mL, Sigma-Aldrich) and 10% Fetal bovine serum (FBS, 7524, Sigma-Aldrich)

* Differentiation test to adipocytes, chondrocytes, and osteocytes passed positive. MSCs specific staining test: positive for CD29, CD44, CD73, CD90, CD105, CD166 and negative for CD14, CD19, CD34, and CD45. Doubling capacity: up to 17 doublings, cells morphology was normal (all the tests performed by ATCC).

Supplementary Table S5: TaqMan gene expression assays

Gene	Cat.nr	Gene	Cat.nr	Gene	Cat.nr
**Human**					
CCL19	HS00171149	TBP	HS00427621	HLA-DRA	Hs00219575
CCL21	HS00989654	CXCL12	HS03676656	IL-2	Hs00174114
CXCL13	HS00757930	IL-7	HS00174202	IL-12	Hs01073447
ICAM1	HS00164932	IL-4	HS00174122	IL-21	Hs00222327
IL-1[illegible]	HS00174097	RANKL	HS00243522	IL-23	Hs00372324
LT[illegible]R	HS00158922	CCR5	HS99999149		
LTβ	HS00242739	CCR7	HS01013469		
TNF-[illegible]	HS00174128	PDPN	HS00366766		
VCAM1	HS01003372	IDO1	Hs00984148		
**Murine**					
CCL19	Mm00839967_g1	LT[illegible]R	Mm00440235_m1	ICAM1	Mm00516023_m1
CXCL13	Mn0444533_m1	LTβ	Mm00434774_g1	VCAM1	Mm01320970_m1
